# Analysis of the 9p21.3 sequence associated with coronary artery disease reveals a tendency for duplication in a CAD patient

**DOI:** 10.18632/oncotarget.24567

**Published:** 2018-02-26

**Authors:** Natalay Kouprina, Mikhail Liskovykh, Nicholas C.O. Lee, Vladimir N. Noskov, Joshua J. Waterfall, Robert L. Walker, Paul S. Meltzer, Eric J. Topol, Vladimir Larionov

**Affiliations:** ^1^ Developmental Therapeutics Branch, National Cancer Institute, Bethesda, MD 20892, USA; ^2^ Genetics Branch, National Cancer Institute, Bethesda, MD 20892, USA; ^3^ The Scripps Translational Science Institute, The Scripps Research Institute and Scripps Health, La Jolla, CA 92037, USA

**Keywords:** TAR-cloning, segmental duplication, genome alterations, 9p21, CAD interval

## Abstract

Tandem segmental duplications (SDs) greater than 10 kb are widespread in complex genomes. They provide material for gene divergence and evolutionary adaptation, while formation of specific *de novo* SDs is a hallmark of cancer and some human diseases. Most SDs map to distinct genomic regions termed ‘duplication blocks’. SDs organization within these blocks is often poorly characterized as they are mosaics of ancestral duplicons juxtaposed with younger duplicons arising from more recent duplication events. Structural and functional analysis of SDs is further hampered as long repetitive DNA structures are underrepresented in existing BAC and YAC libraries. We applied Transformation-Associated Recombination (TAR) cloning, a versatile technique for large DNA manipulation, to selectively isolate the coronary artery disease (CAD) interval sequence within the 9p21.3 chromosome locus from a patient with coronary artery disease and normal individuals. Four tandem head-to-tail duplicons, each ∼50 kb long, were recovered in the patient but not in normal individuals. Sequence analysis revealed that the repeats varied by 10-15 SNPs between each other and by 82 SNPs between the human genome sequence (version hg19). SNPs polymorphism within the junctions between repeats allowed two junction types to be distinguished, Type 1 and Type 2, which were found at a 2:1 ratio. The junction sequences contained an Alu element, a sequence previously shown to play a role in duplication. Knowledge of structural variation in the CAD interval from more patients could help link this locus to cardiovascular diseases susceptibility, and maybe relevant to other cases of regional amplification, including cancer.

## INTRODUCTION

**Figure 1:**
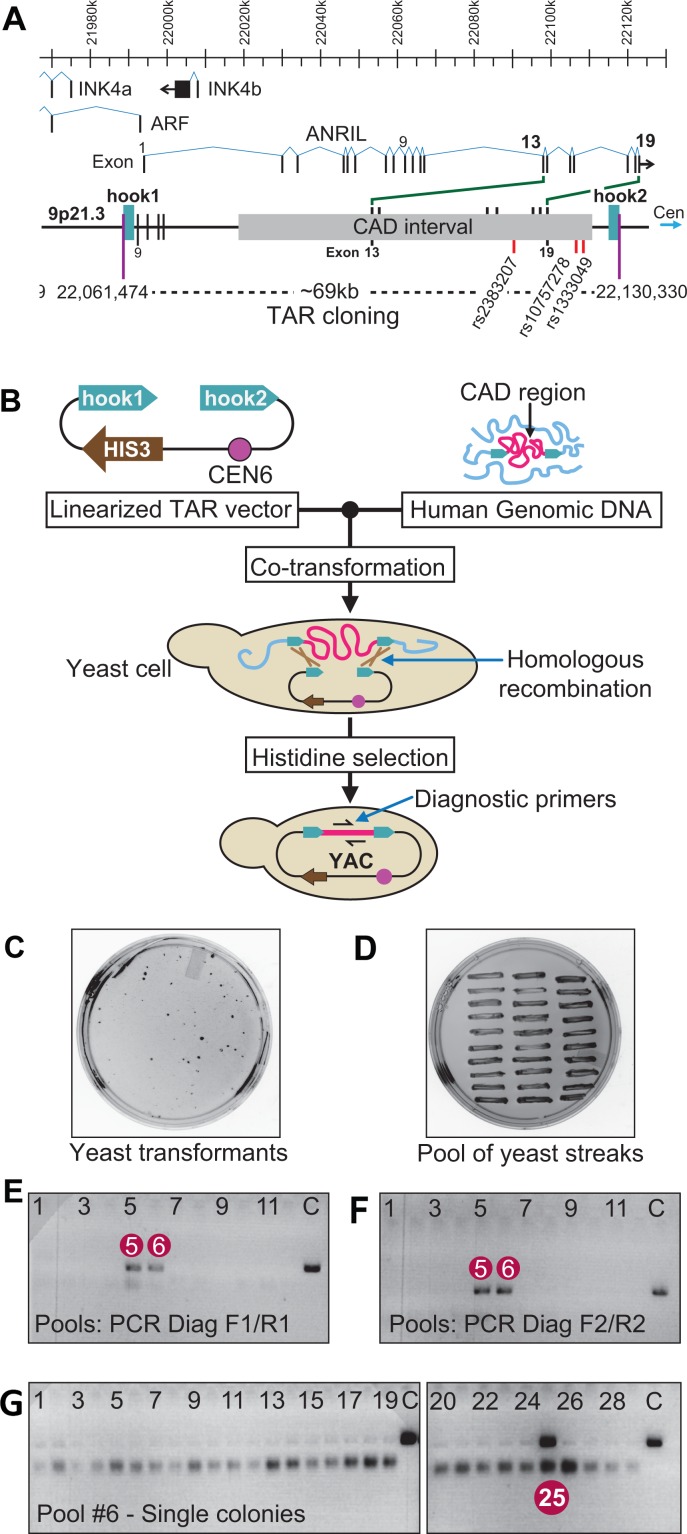
TAR cloning of the CAD interval at 9p21.3 from a patient with the coronary artery disease **(A)** A scheme of organization of the 9p21.3 region. Positions of three known genes, ARF, INK4a and INK4b, as well as a long noncoding RNA, ANRIL, are shown. ANRIL transcript covering 126 kb includes the CAD interval. ANRIL consists of 19 exons. Exons 13-19 are within the CAD interval. Positions of three SNPs, rs10757278, rs1333049 and rs2383207, specific to the mutated allele of the CO87 patient are shown. Positions of the targeted sequences (hooks) chosen for TAR cloning are indicated (human genome sequence; version hg19). **(B)** The diagram showing a general scheme of TAR cloning of a region of interest (in red) from total genomic DNA (in blue) with a linearized TAR vector containing a yeast selectable marker HIS3, centromeric sequence from chromosome 6 (CEN6) and two unique targeting sequences (hook1 and hook2) homologous to 5’ and 3’ ends of the targeted region. After co-transformation into yeast *Saccharomyces cerevisiae*, recombination between targeting sequences in the vector and the targeted sequences of the genomic DNA fragment leads to the rescue of the fragment as a circular YAC (yeast artificial chromosome) molecule. For TAR cloning experiments, the vector DNA is linearized by a unique endonuclease located between the hooks to expose targeting sequences. **(C)** A representative His^−^ plate with yeast His^+^ transformants and **(D)** the plate with 30 streaked randomly chosen His^+^ transformants. **(E)**, **(F)** Analysis of 11 pools of yeast transformants by PCR for the presence of the CAD region using two pairs of diagnostic primers, F1/R1 and F2/R2). Pools #5 and #6 are positive for diagnostic PCR. **(G)** Analysis of 30 individual His^+^ transformants from pool # 6. Clone #25 is positive for the CAD interval. C-control PCR with human genomic DNA.

A growing number of genome-wide associations studies (GWAS) have identified specific regions of the human genome with a strong non-random correlation to complex human traits such as predispositions to diseases [1]. One of such regions has been identified on the INK4b-ARF-INK4a gene cluster located on the human chromosome 9p21.3 (Figure 1A). This region is tightly related with the increase of coronary artery disease (CAD), myocardial infarction [1, 2], ischemic stroke [2, 3] and aortic aneurysm [4]. Recent GWAS has linked single nucleotide polymorphisms (SNPs) at 9p21.3 to CAD and other related similar conditions [2, 3, 5–15]. These associations have been confirmed in multiple independent studies [2, 3, 5–15]. While causal variants within 9p21.3 have yet to be identified, the risk-associated SNPs cluster together within a ∼60 kb region, roughly 100 kb centromeric to the INK4/ARF locus [16]. This 60 kb sequence, named as the CAD interval [17, 18], does not contain protein coding genes.

In addition to CAD, there is a strong correlation of polymorphism at 9p21.3 with predispositions to other diseases, including type II diabetes [19, 20], glioma [21–24], esophageal squamous cell carcinoma (ESCC) [25] and glaucoma [26, 27]. In some cases, such correlation may be linked to mutations in three tumor suppressor genes within the INK4/ARF locus, ARF, INK4b and INK4a. These genes play a central role in cell-cycle arrest, thus affecting key cellular processes such as senescence, apoptosis, and stem cells self-renewal [28, 29]. This locus also contains a fourth gene, MTAP, which has annotated exons overlapping the INK4/ARF locus [30]. MTAP catalyzes the phosphorylation of 5’ methyladenosine in the polyamine pathway, and it has also been associated with carcinogenesis [31].

However, GWAS analysis more frequently correlates predisposition to disorders with SNPs mapped to intergenic or non-coding regions rather than to sequences corresponding to annotated genes [32]. At present, it is presumed that some diseases risk caused by SNPs at 9p21.3 act through the long non-coding RNA CDKN2B-AS1, commonly referred to as the Antisense Non-coding RNA in the INK4 locus (ANRIL). ANRIL was first identified within a 403 kb germ-line deletion in a family with a history of melanoma and neural system tumors [33]. ANRIL is transcribed as a 3.8-kb-long non-coding RNA from the short arm of human chromosome 9 at the p21.3 locus that overlaps a critical region encompassing three major tumor suppressor loci juxtaposed to the INK4b-ARF-INK4a gene cluster and the MTAP gene [34]. It is transcribed in the direction opposite to the INK4b-ARF-INK4a gene cluster and shares a bidirectional promoter with ARF. The 3’ end of ANRIL is terminated at the very end of the CAD interval. Based upon EST assembly, ANRIL has 19 exons with no identified open reading frame. Although cloning a full-length version of the predicted transcript has proven to be difficult, a growing number of alternatively spliced ANRIL transcripts, including circular forms, have recently been reported in the literature [16, 35]. Prior work has shown that long, non-coding RNAs such as Xist, Kcnq1ot1 and HOTAIR can repress genes in cis- or trans- through interaction with Polycomb group (PcG) complexes [36–40]. It has also been postulated that ANRIL could play a similar role in PcG-mediated repression of the INK4b-ARF-INK4a gene cluster [41].

In this work, we focused on the analysis of structural variations within the CAD interval sequence using different approaches, including re-isolation of this region by the transformation-associated recombination (TAR) cloning technique [42, 43, 44, 45]. TAR cloning represents a unique tool for selective isolation and manipulation of large DNA molecules. The technique exploits a high level of homologous recombination in the yeast *Sacharomyces cerevisiae*. So far, TAR cloning is the only method available to selectively recover chromosomal segments or genes up to 350 kb in length from simple and complex genomes, including human [42, 43, 44, 45]. In the post-genomic era, TAR cloning has found many applications for functional and structural genomics. For example, it can be used to isolate rearranged chromosomal regions, such as translocations and inversions, from patients and model organisms. TAR cloning allows the assembly and cloning of entire microbe genomes up to several Mb as well as engineering of large metabolic pathways [45].

Here, we used TAR cloning for isolation of the CAD region from a somatic peripheral blood mononuclear cells line derived from a coronary artery disease patient with unique mutations within the CAD interval. As a control, the CAD region was TAR-cloned from blood cells of normal individuals. Our analysis of TAR isolates revealed a previously unknown amplified 50 kb sequence corresponding to the CAD interval in the patient but not in normal individuals. This observation may be important for GWAS studies aiming to link SNPs near the INK4b-ARF-INK4a gene cluster to susceptibility to cardiovascular diseases.

## RESULTS

### TAR-isolation of the CAD interval sequence from the cell line derived from a patient with the coronary artery disease

Figure 1A illustrates the organization of the INK4b-ARF-INK4a gene cluster at 9p21.3 with the positions of the ANRIL ncRNA and the CAD interval carrying high-risk SNPs. The region chosen for analysis includes the CAD interval with flanking sequences. Figure 1B illustrates a diagram of TAR cloning of the CAD-containing region from the patient derived C087 cell line. The TAR vectors 9p21-1 and 9p21-2 (Supplementary Figure 1) were constructed and used for TAR cloning experiments. The vectors contained a yeast selectable marker HIS3, a yeast centromere from *Saccharomyces cerevisiae* chromosome 6 (CEN6) and two unique targeting sequences (hooks) homologous to the 5’ and 3’ ends of the targeted genomic region. Vector 9p21-1 was constructed by insertion of two DNA targeting hooks, 171-bp and 209-bp long, corresponding respectively to positions 22,062,540 to 22,062,711 and 22,129,014 to 22,129,313 on human chromosome 9 (human genome sequence; version hg19). The expected size of the captured genomic fragment is 66,763 bp. As for vector 9p21-2, the hooks used were 185-bp and 242-bp long, corresponding to positions 22,061,474 to 22,061,658 and 22,130,089 to 22,130,330 on the chromosome 9 (version hg19) The expected size of the 9p21-2 targeted genomic fragment is 68,856 bp.

After transformation of a TAR vector and genomic DNA into yeast *S. cerevisiae* cells, recombination between the hooks in the vector and the targeted genomic sequences leads to the rescue of the target region as a circular TAR/YAC (Yeast Artificial Chromosome) molecule (Supplementary Figure 2A) [42, 43]. To identify the desired region-containing clones, yeast transformants (Figure 1C) were combined into pools (Figure 1D) and then examined with PCR reactions using diagnostic primers, F1/R1 and F2/R2 (Supplementary Table 1). These primers are not present in the TAR vector but are specific to the targeted genomic region. As an example, Figure 1E and 1F illustrates the PCR screening of 11 pools of transformants obtained by the 9p21-2 vector. Pools #5 and #6 are positive for diagnostic primers. Figure 1G illustrates the PCR analysis of 29 transformants of pool #6. Individual transformant #25 is positive for diagnostic primers. Using both vectors, eight CAD-region-positive TAR/YAC clones were identified and used for further analysis.

### Physical analysis of TAR clones isolated from C087 cells suggests the presence of segmental duplication

**Figure 2:**
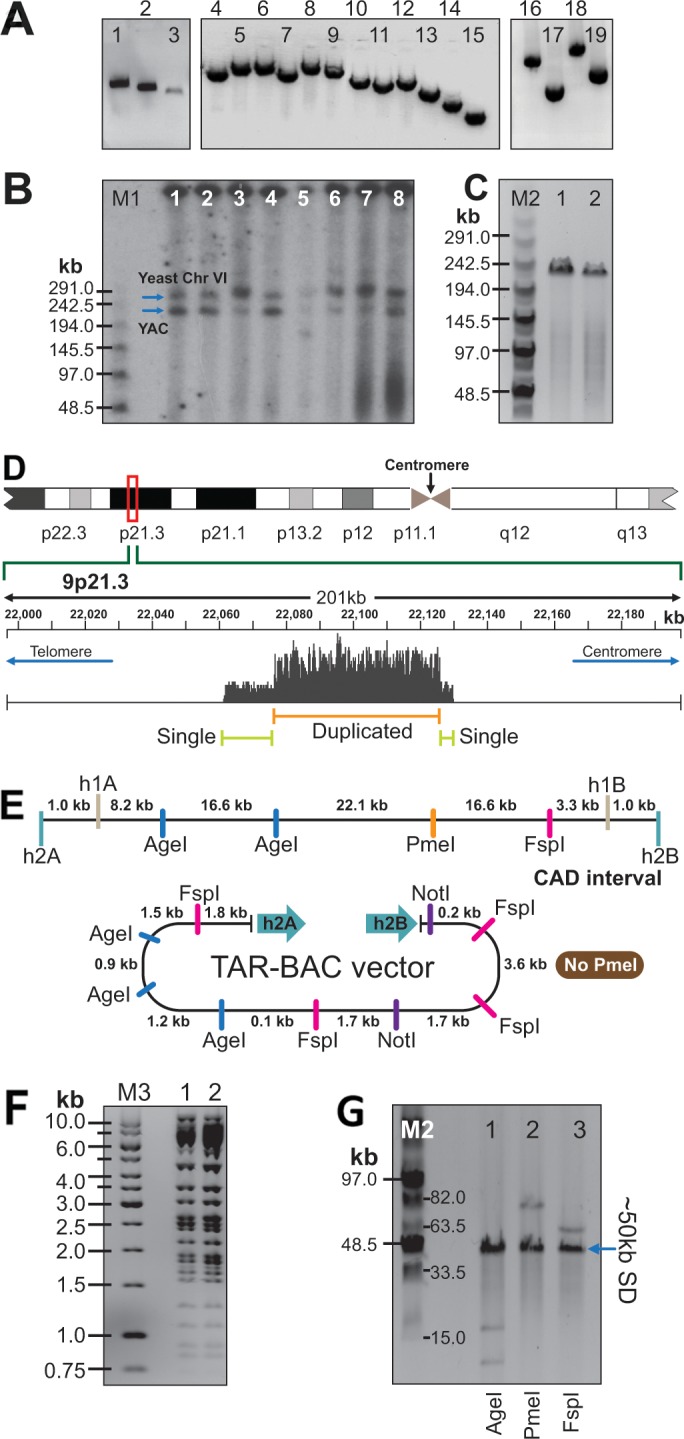
Physical analyses of the CAD-containing TAR/YAC and BAC clones isolated from the C087 patient cell line **(A)** PCR analysis of a YAC clone by a set of the overlapping primers (Supplementary Table 1) covering the entire CAD region (positions 22,061,474 to 22,130,330 on the chromosome 9 in hg19). Lane 1 corresponds to New41F/New41R; lane 2 – New41F/41R; lane 3 – 41F/New41R; lane 4 - 17F/20R; lane 5 - 20F/27R; lane 6 – 33R/27F; lane 7- 46R/42F; lane 8 - 51R/46F; lane 9 – 51F/59R; lane 10 – 33F/35R; lane 11 – 33F/38R; lane 12 – 38F/40R; lane 13 – 40F/41R; lane 14 – 42F/42R; lane 15 – New41F/New41R; lane 16 – 6500F/13R; lane 17 – 13F/17R; lane 18 -6500F/17R; lane 19 – 38F/41R. **(B)** Size of the TAR/YAC-cloned material. NotI-digested DNA isolated from eight independent TAR/YAC clones was separated by CHEF gel electrophoresis and hybridized with the CEN6 probe. Arrows indicate an yeast centromere from chromosome 6 and linearized YAC molecules. **(C)** Size of the TAR/BAC clones. Two BAC DNAs (lane 1 - 9p21-2 vector; lane 2 – 9p21.1 vector) were digested by NotI, separated by CHEF, and visualized with ethidium bromide. The size of the bands is ∼230 kb. **(D)** Snapshot of the region from the Integrative Genomics Viewer (IGV) showing the coverage of reads obtained from sequencing of the C087 BAC A218. As seen, the duplicated region corresponds to positions 22076300 to 22126800 on chromosome 9 (hg19). **(E)** Schemes of the CAD region at 9p21.3 and the TAR vector after retrofitting by BRV1 vector. **(F)** HindIII digestion profiles of two BACs. **(G)** Analysis of the repeated sequence in the BAC clone A218 containing the CAD region. BAC DNA were digested either AgeI (lane 1) or PmeI (lane 2) or FspI (lane 3). M1 - CHEF DNA Size Lambda Ladder (BIO-RAD); M2 – Midrange 1 PFG Marker (NEB). M3 – GeneRuler 1 kb DNA Ladder (Fermentes).

TAR isolates containing the CAD region were characterized in a Yeast Artificial Chromosome (YAC) and a Bacteria Artificial Chromosome (BAC) form. The retrofitting vector BRV1 was used to convert YAC clones into BACs [44]. Details are described in MATERIALS AND METHODS and in Supplementary Figure 2B. Several approaches were taken to characterize the TAR-cloned material. Firstly, to prove the presence of the predicted genomic sequence in YAC isolates, yeast clones were examined by PCR with overlapping pairs of primers (Supplementary Table 1) that cover the entire CAD interval. As seen, this region is present in the TAR clones (Figure 2A). Secondly, to check the size of the cloned material, yeast chromosomal-size DNAs were exposed to a low dose of γ-rays to linearize circular YAC molecules, separated by clamped homogeneous electrical field electrophoresis (CHEF), blotted and hybridized with the yeast CEN6 probe (Supplementary Table 1). Unexpectedly, the size of the YACs was much bigger than predicted (∼230 kb versus ∼67/69 kb) (Figure 2B). After conversion of YACs into BACs, we analyzed the TAR/BAC clones. To check the size of BACs, they were digested by NotI, separated by CHEF and stained with ethidium bromide. The size of BACs corresponded to the size of YAC isolates (∼230 kb) (Figure 2C). To demonstrate the identity of sequences in BAC clones, DNA isolated from two randomly selected BACs was digested by HindIII. The restriction profiles of the BACs were indistinguishable from each other (Figure 2F).

The larger size of TAR isolates may be explained either by the presence of duplications within the CAD interval or by the presence of non-annotated sequences in this region. To clarify this, one of the BAC clones (A218) was used for deep paired-end sequencing. This identified 82 SNPs that differed from the human genome sequence (version hg19) (Supplementary Table 2) but no evidence for large insertions of novel sequence. Surprisingly, analysis of depth of coverage as well as discordant read pair alignments suggested copy number gain of an approximately 50 kb internal region (Figure 2D) arranged in a tandem head-to-tail orientation. To confirm that the CAD-carrying clones contain segmental duplications, DNA from the BAC A218 was digested either by AgeI or PmeI or FspI endonucleases that cuts once within the proposed 50 kb repeat (Figure 2E). After digestions, it was clearly seen that the BAC insert has approximately 50 kb duplicated region (Figure 2G). For example, digestion by AgeI (Figure 2G; lane 1) produces an intense ∼50 kb band and two other minor expected bands, i.e. a 16.6 kb band derived from the 5’ end of the CAD interval and a smaller band of 12.5 kb derived from the vector part and the region between the hook and the first AgeI site (Figure 2E). This suggests that a 50 kb sequence is repeated four times (50 kb x 4 plus 16.6 kb plus 12.5 kb = 229.1 kb).

**Figure 3:**
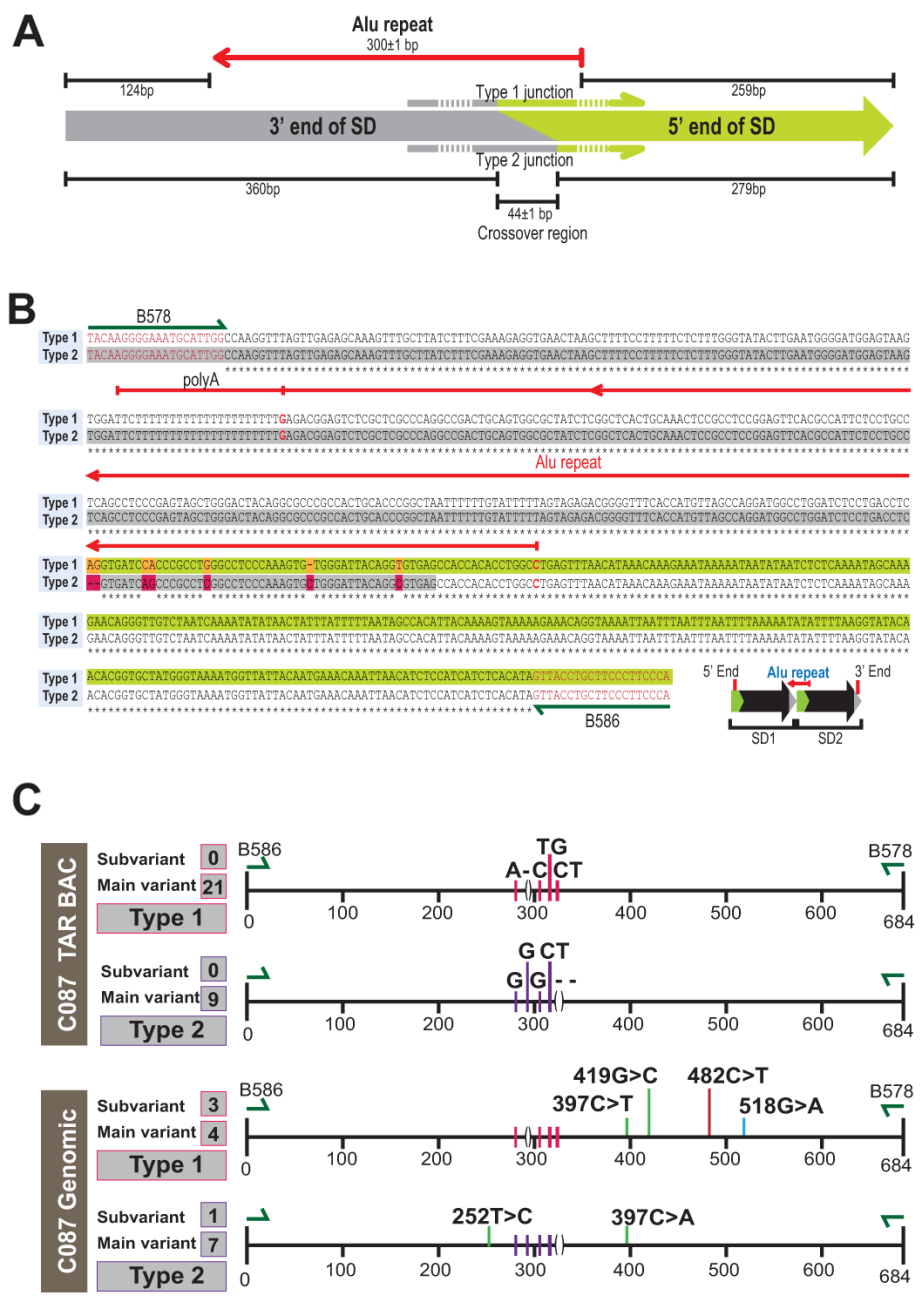
Junction between segmental duplications **(A)** Scheme of SD junction depicting the 3’ and 5’ ends of the SD, the relative position of the Alu repeat and the crossover region found in Type 1 and Type 2 junction sequences. **(B, C)** Sequence alignment of two major types of junction amplified by B578/B586 primers (nucleotides in red). Red arrow is the Alu repeat. Nucleotide polymorphisms unique to each type of junction are depicted by colored boxes.

To prove the presence of the predicted junction from the deep sequencing (Figure 2D), we designed a specific pair of forward and reverse primers, B586/B578, corresponding to the very beginning and end of the proposed 50 kb repeat (Supplementary Table 1). These primers amplified a 684 bp product from BAC DNA. Sequencing of these PCR products revealed the following structure, i.e. 124 bp (positions 22126447 to 22126570 in hg19) corresponding to the end of the proposed duplication and 259 bp (positions 22076352 to 22076610 in hg19) that corresponds to the beginning of the repeat. Within the PCR products, these sequences are separated by the 301 bp Alu element (Figure 3A and Supplementary Table 3). SNPs polymorphism within the junction sequences allowed us to distinguish two types of junctions, Type 1 and Type 2 (Figure 3B and 3C) that were found at an approximate ratio of 2:1. In addition, we designed other pairs of primers further upstream and downstream of B586/B578 primers (see “Extended PCR junction” in Supplementary Table 1). These primers also gave the junction products (Supplementary Figure 3). Their sequencing confirmed the proposed junction between repeats.

To summarize, our results suggest that the C087 TAR isolates contain a segmental duplication (SD) of 50,635bp in size (positions 22076220 to 22126855 in hg19).

### The structure of the CAD interval derived from a patient with the coronary artery disease

**Figure 4:**
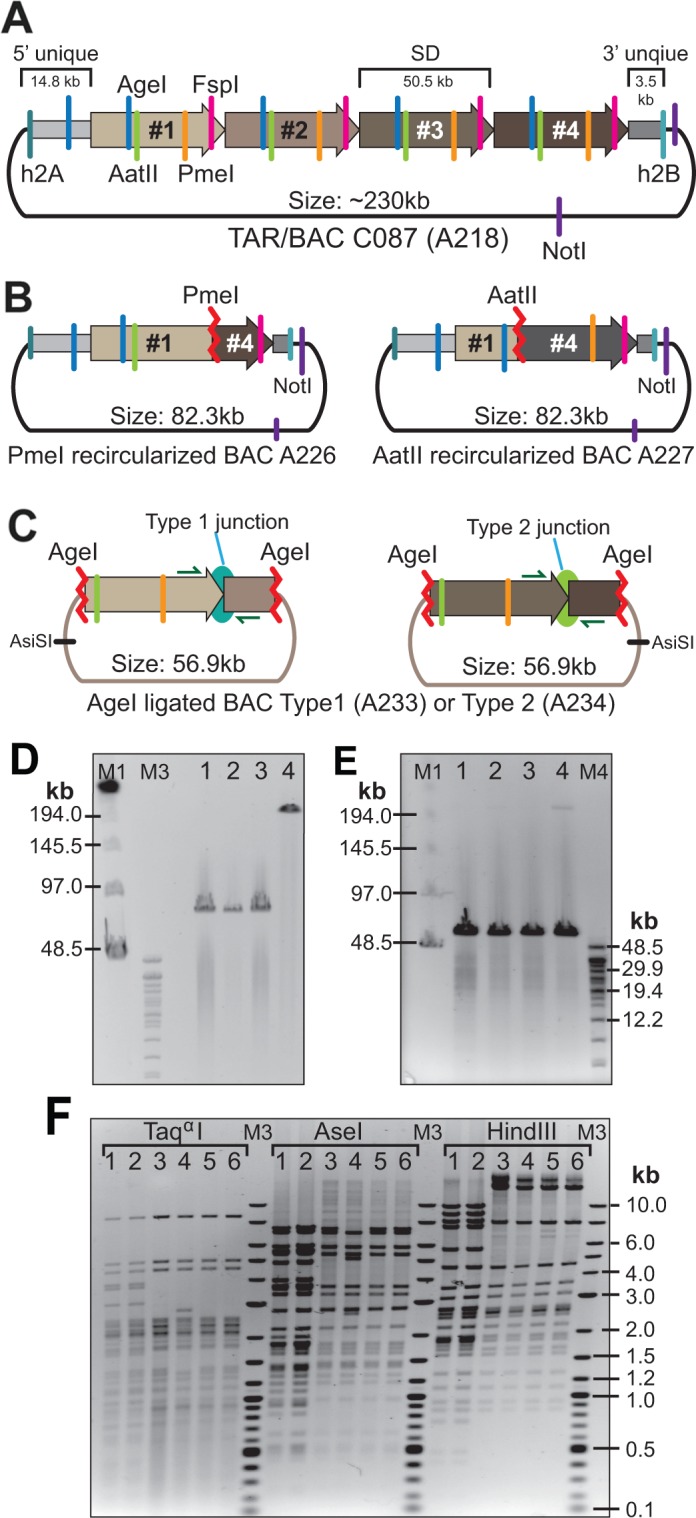
Confirmation of the presence of SDs in the CAD-containing C087 TAR/BAC clone **(A)** A predicted structure of BAC A218. **(B)** The structure of A226 and A227 BACs obtained after PmeI or AatII digestion of BAC A218 and followed by re-ligation. **(C)** The structure of A233 and A234 BACs obtained after AgeI digestion of BAC A218 and followed by capture into V231 vector. **(D)** CHEF analysis of one isolate of NotI-digested BAC A226 (lane 1) and two isolates of BAC A227 (lanes 2, 3). Lane 4 corresponds to NotI-digested BAC A218. **(E)** Lanes 1, 2 correspond to two isolates of AsiSI-digested BAC A233. Lanes 3, 4 correspond to two isolates of AsiSI-digested BAC A234. **(F)** Restriction profiles of A226, A227, A233 and A234 BACs after digestion either by Taqα1 or AseI or HindIII. Lane 1 corresponds to BAC A226. Lane 2 corresponds to BAC A227. Lanes 3, 4 correspond to two isolates of BAC A233. Lanes 5, 6 correspond to two isolates of BAC A234.

Based on the data described above, we predicted the physical structure of the CAD interval within the TAR isolates (Supplementary Figure 4). To prove this structure, we sub-cloned the restriction digest fragments of BAC A218 (Figure 4A) and sequenced them.

Firstly, if the proposed map is correct, complete digestion of BAC DNA either by PmeI or AatII followed by re-ligation should produce a BAC containing a hybrid ∼50 kb unit plus flanking regions containing hooks (Figure 4A). So, BAC A218 was digested, re-ligated and electroporated into *E. coli* cells. The Cm^R^ colonies were screened by PCR with the primers specific for the yeast HIS3 gene (a positive control), for the sequences around the PmeI site (a positive control) and for the predicted junction sequence that should not present in these BACs (a negative control) (Supplementary Table 1). BAC DNA from appropriate colonies (Figure 4B) was isolated, digested with NotI and run on CHEF. All the BACs have the expected size of ∼82 kb (Figure 4D; lanes 1, 2, 3). Two BACs, A226 after PmeI digestion and A227 after AatII digestion, were chosen for deep sequencing.

Secondly, to isolate the internal repeats, BAC A218 was digested by AgeI and run on CHEF. The fragments of ∼50 kb in size were isolated from the gel and ligated into the vector V231 (Supplementary Figure 5). The Cm^R^ colonies were screened by PCR using the primers specific for the junction between proposed SDs (Supplementary Table 1). BACs with Type 1 (A233) and Type 2 (A234) junction were identified (Figure 4C). Two BACs of each junction type were isolated, digested with AsiSI that has a unique site in the V231 vector and run on CHEF. The predicted ∼57 kb single bands were observed (Figure 4E).

Thirdly, to confirm the identical structure of the re-cloned fragments, BACs A226, A227, A233 and A234 were digested either by Taqα1 or AseI or HindIII (Figure 4F). The restriction profiles of BACs A233 and A234 were identical to each other (Figure 4F; lanes 3, 4, 5, 6). The restriction profiles of BACs A226 (PmeI digested) and A227 (AatII digested) were also identical to each other (Figure 4F; lanes 1, 2) and shared similarities with the banding profiles of BACs A233 and A234. BAC A233 (Type 1) and BAC A234 (Type 2) were chosen for deep sequencing.

Sequence analysis of the BACs confirmed that the CAD-containing TAR-cloned region consists of almost identical ∼50 kb repeat units. Notably that each unit contains three characteristic SNPs, rs10757278, rs1333049 and rs2383207, that are specific to the risk allele of the CO87 patient (Supplementary Figure 6).

### Analysis of TAR isolates from normal human cells did not reveal the presence of duplicated region within the CAD interval

**Figure 5:**
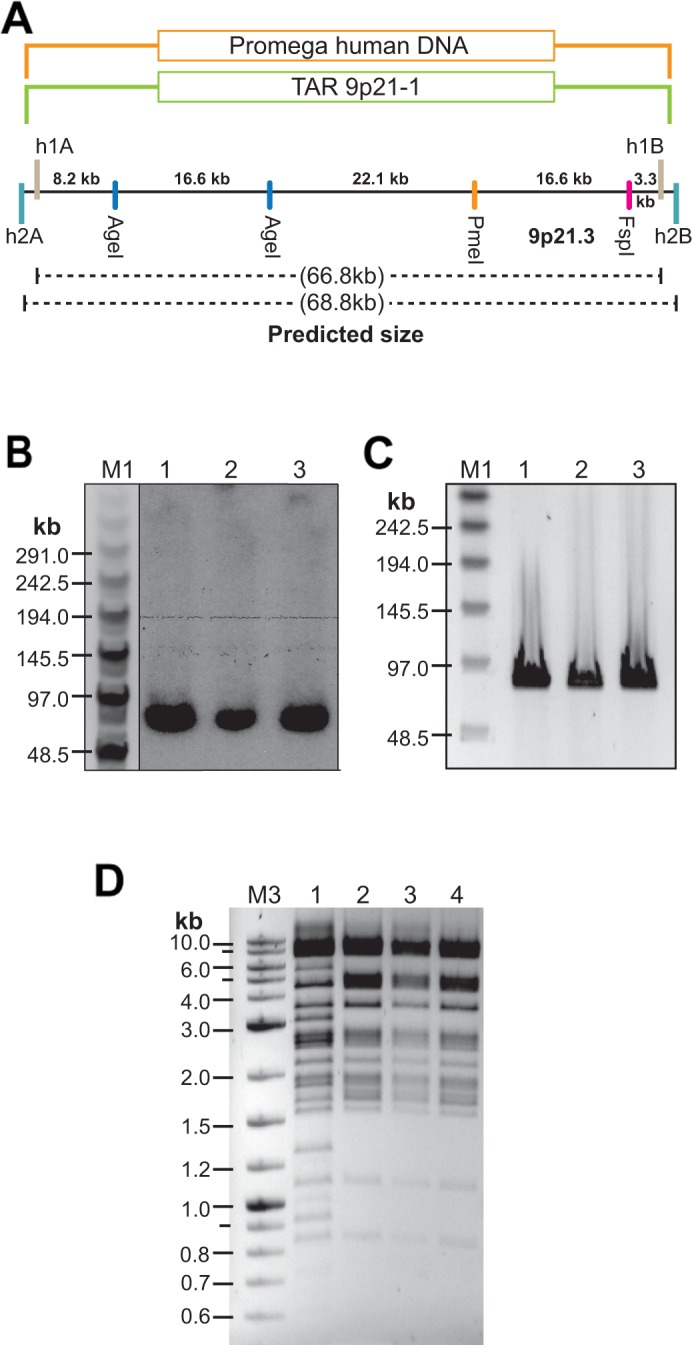
TAR cloning and physical analysis of the CAD-containing TAR/YAC clones isolated from genomic DNA of normal individuals **(A)** A scheme of TAR cloning of the CAD region from DNA isolated from Promega genomic DNA by 9p21-1 TAR vector. h1A and h1B are targeting hooks for 9p21-1 TAR vector. h2A and h2B are targeting hooks for 9p21-2 TAR vector. **(B)** Southern analysis of the TAR/YAC isolates. Genomic DNA from three independent YAC clones was digested by NotI, separated by CHEF, and blot-hybridized with a 862 bp specific probe (positions on chromosome 9: 22084459-22085320 in human genome sequence; version hg19). The size of the predicted cloned material is ∼66.8 kb. **(C)** CHEF analysis of NotI-digested three BAC isolates (lanes 1, 2 correspond to BAC clones obtained from a YAC clone #1; lane 3 corresponds to a BAC obtained from a YAC clone #2). **(D)** HindIII digestion profiles of BACs from the C087 patient and normal individuals. Lane 1 corresponds to genomic DNA isolated from the A218 BAC clone of the C087 patient containing four 50 kb repeat units; Lanes 2 – 4 correspond to genomic DNA isolated from BAC clones of normal genomic DNA (Promega). M1 - CHEF DNA Size Lambda Ladder (BIO-RAD); M2 - Quick-Load 2-Log DNA Marker (BioLabs).

We used the TAR 9p21-1 vector (Supplementary Figure 1) to TAR clone the CAD region from the normal human DNAs (Promega). Three CAD-positive TAR/YAC clones were identified and used for further analysis. The presence of the predicted genomic sequences in YAC isolates was confirmed by PCR with pairs of overlapping primers (Supplementary Table 1 and Supplementary Figure 7). To check the size of the cloned material in yeast cells, three YACs were linearized by NotI, separated by CHEF and blot-hybridized with a specific probe (Supplementary Table 1). The size of the YAC inserts was as expected (Figure 5A). After conversion of YACs into a BAC form (Supplementary Figure 2B), the BAC molecules were moved into bacterial cells and analyzed. To check the size of BACs, they were digested by NotI, separated by CHEF and stained with ethidium bromide. The size of BACs corresponded to the size of YAC isolates (Figure 5B and 5C). To demonstrate the identity of sequences in the BAC clones from a patient and normal individuals, DNA isolated from BACs was digested by HindIII (Figure 5D). As expected, the restriction profiles of the BACs were almost indistinguishable from each other.

To confirm that duplication observed in TAR clones isolated from the C087 cells was not due to amplification of the cloned material in yeast cells, we performed a control experiment. A BAC A226 containing a single copy of the CAD region was re-transformed into yeast cells. After that, genomic DNA from 10 His^+^ transformants were isolated, NotI digested, separated by CHEF and blot-hybridized with a specific probe of 862 bp in size (positions on chromosome 9: 22084459 to 2085320 in hg19) (Supplementary Table 1). As seen from Supplementary Figure 8, all transformants have the expected size, which proves an absence of amplification of this region in yeast cells. As expected, the TAR isolates did not contain SNPs, rs10757278, rs1333049 and rs2383207, which are specific to the risk allele of the CAD patient (data not shown).

### Population analysis of the CAD interval at 9p21.3 in the human genomes did not reveal repeated sequences

To check whether the copy number of *SD at 9p21.3* is variable in human population, we applied quantitative real-time PCR to analyze 181 DNA samples from normal individuals. Copy number of two unique sequences, 144 bp and 137 bp in size, at the beginning and the end of SD (positions on chromosome 9: 22078776 to 22078919 and 22117414 to 22117550 in hg19) was determined using two pairs of specific primers (Supplementary Table 1). As a control for a single copy region, we used a unique 87 bp sequence outside of SDs (positions on chromosome 9: 22062099 to 22062185 in human genome sequence version hg19) and the RNAaseP gene that allowed the reactions to be calibrated and reproducible results to be obtained. Table 1 summarizes the results of analysis. As seen, all samples contain one copy of the CAD interval sequence. In addition, Droplet digital PCR re-confirmed these results (Supplementary Table 4). Thus, more than one copy of SD is not very common in human population (less than 1%).

**Table 1:** Analysis of the number of SD at the CAD region in normal individuals by qPCR^*^

Population	Total analyzed number	Samples with 1 copy	Samples with 2 copy	Samples with 4 copy
Korean	52	52	0	0
Italian	46	46	0	0
Caucasian	83	83	0	0
Patient C087	1	0	0	4

^*^The number is given per haploid genome.

## DISCUSSION

In the past years, an emerging group of human genetic diseases have been described that result from DNA rearrangements rather than from single nucleotide changes. Such conditions have been referred to as genomic disorders. The predominant molecular mechanism underlying some of such rearrangements [(also known as segmental duplications (SDs)] is nonallelic homologous recombination (NAHR) utilizing the repeats as substrates. These higher-order genomic architectural features usually span from ∼10 kb up to hundreds of kilobases of genomic DNA where repeats/duplications share >90% sequence identity. Notably, 52% of the remaining gaps in the reference haploid human genome, refractory regions to all techniques available at the moment, are sequences consisting of SDs. In humans, copy-number variations (CNVs) of such repeats have been implicated in common traits such as neuropathy, hypertension, color blindness, infertility, and behavioral traits, including autism and schizophrenia, as well as disease susceptibility to HIV, lupus nephritis, psoriasis and Parkinson's disease [46–48].

In this study, comprehensive analysis of the CAD interval at 9p21.3 locus, where SNPs associated with coronary artery disease (CAD) were mapped [1–4], revealed a previously un-annotated duplication of ∼50 kb in size in the human peripheral blood mononuclear cell line derived from a patient with CAD. Based on the sequence analysis of TAR isolates, the CAD region in this cell line contains four 50 kb units that are organized as tandem repeats. An Alu repeat is present in the junction sequence between the units. It is worth noting that the role of Alu in formation of duplications has been proposed for this type of SINE elements [49]. Quantitative real-time PCR and Droplet digital PCR analyses showed no amplification of this region in the limited number of normal human individuals (approximately 200 DNA samples were screened that means that such duplication may represents less than 1% in human population).

This CAD duplication has also not been previously detected in human genome studies. This may be explained by several reasons. Firstly, although computational methods have been developed to identify duplicated sequences independently on the genome assembly and other experimental methods like FISH and array comparative genomic hybridization (array-CGH) have been used to validate and explore the distribution and organization of such sequences [20], these methods work well for relatively short SDs (size between 1 kb and 10 kb). Identification of large SDs by these methods remains a challenge. Secondly, the CAD region does not contain any open reading frames that are easily identified by Next-Generation Sequencing. Thirdly, there is a relatively high density of SINE and LINE repeats within the region. This makes it a problem to assemble the duplicated sequences using the multitude short reads obtained from Next-Generation Genome Sequencing. The identification of SDs within the SPANX-B locus at Xq27 is a good example of such problems. It is worth noting that the presence of SDs within the SPANX-B locus at Xq27 was determined only by direct TAR cloning of the SPANX-B region from human genomes [50, 51]. Fourthly, some duplicated regions are poorly represented in BAC libraries and subsequently become underrepresented if these libraries are used to build genome maps. One potential reason for the poor representation of some duplicated region in BAC libraries is the absence of appropriate restriction site for digestion of genomic DNA before ligation into a BAC vector. Moreover, some DNA fragments carrying large, homologous repeats are sometimes structurally unstable in yeast and bacteria, for example the SPANX-B region [50, 51]. Finally, it is a matter of data analysis. The 150 kb TAR/BAC obtained from the patient in this study was sequenced using Next-Generation Sequencing technology. Initial analysis reported and assembled a single 50 kb sequence corresponding to the known human genome sequence (version hg19). This 50 kb reported length did not match the physical length that we knew the TAR/BAC to be (150 kb). The duplication was only detected within the data when we specifically called for a comparison between the number of sequence reads in the duplicated area versus the BAC backbone.

Nevertheless, some indirect hints for the presence of the CAD duplication came from the recent work describing novel linear and circular forms of a long non-coding RNA, ANRIL [16]. This RNA consists of 19 exons. The last exons (exons 13-19) are mapped to the 50 kb CAD duplication (Figure 1A). In some forms, the order of exons is abnormal, e.g. exons 16-17-18-19-13-14-15-16. Such exon order may be explained not only by existence of a circular form but also by a splicing of a transcript overlapping two neighboring 50 kb duplicated regions within the CAD interval.

Further studies that would integrate GWAS data with those obtained from detailed genomic analysis of multiple tissues by TAR cloning in combination with qPCR are required to understand the complex genetic regulation of the 9p21.3 region and its role in the development of cardiovascular disease. The discovery of SDs within the CAD interval in the human peripheral blood mononuclear cells derived from a patient with the coronary artery disease suggests that the number and/or divergence of duplications may be an additional factor affecting the development of this disease. Though the CAD interval does not contain protein coding genes but it does encode a long non-coding RNA (ANRIL). It is presumed that some disease risk factor acts through long non-coding RNAs. Thus, the number of duplications may affect the level of ANRIL expression or splicing of this non-coding RNA located within the duplicated region that may determine predisposition to CAD. It is worth noting that specific SNPs linked to CAD are located within the duplicated region and are presented in the spliced products. This is very similar to the 12 kb repeat at Xq27, within which the SPANX-B gene is located. The number of this repeat varies from 1 up to 7 copies in different individuals [51]. It is known that SPANX-B is expressed only in testis. So, it may be assumed that the change of SPANX-B expression due to its copy number is linked to predisposition to prostate cancer or infertility. Thus, only a direct analysis of ANRIL expression in multiple CAD patients will give a final answer of involvement of the found duplication in this particular locus in cardiac disease.

To summarize, before a conclusive link between the SDs and the cardiovascular diseases can be made, further analysis is required on the CAD interval in more patients with coronary artery disease and in the human population, using the TAR cloning technique in combination with qPCR or Droplet digital PCR developed in this work. Moreover, our finding may be interesting to other labs working with the 9p21.3 region tightly related with other diseases such as myocardial infarction, ischemic stroke and aortic aneurysm.

## MATERIALS AND METHODS

### Cell lines and media

The cell line C087 was derived from somatic cells of a patient with unique mutations within the CAD (coronary artery disease) interval. The cells were grown in mTeSR^TM^1 medium (Stemcells^TM^ Technologies). The patient C087 is known to have coronary artery disease, and is a male, with an age of 49 years old at the time of PBMC isolation. The patient carries risk alleles at 9p21.3, i.e. rs10757278, rs1333049, and rs2383207.

### Genomic DNA

Human DNA purified from blood cells of normal individuals was purchased from Promega (Cat. No. G3041). Genomic DNA from normal human individuals was used for qPCR and Droplet digital PCR. An additional 66 DNA samples were purchased from Coriell Institute for Medical Research. More DNA samples were obtained from Dr. Joanna Schluetker (Institute of Medical Technology, University of Tampere, Finland). A detailed description of these samples is presented elsewhere [50, 51].

### Construction of TAR cloning vectors

The transformation-associated recombination (TAR) vectors, 9p21-1 and 9p21-2, were constructed using the basic vector pVC604 containing a yeast selectable marker HIS3 and a centromere from chromosome 6 [43]. To construct TAR vector 9p21-1, the 171-bp XhoI-ClaI (hook1A) and 209-bp ClaI-SpeI (hook1B) fragments corresponding to 5′ and 3′ regions of the CAD region were inserted into the polylinker of pVC604. The 5′ and 3′ targeting sequences of the vector 9p21-1 were designed based on the available information (hg19) and correspond to positions 22,062,540 to 22,062,711 and 22,129,014 to 22,129,313 on the chromosome 9 sequence. The expected size of the targeted genomic fragment is 66,763 bp. To construct TAR vector 9p21-2, the 185-bp XhoI-ClaI (hook2A) and 242-bp ClaI-SpeI (hook2B) fragments corresponding to 5′ and 3′ regions of the CAD region were inserted into the polylinker of pVC604. The 5′ and 3′ targeting sequences of the vector 9p21-2 were designed based on the available information (hg19) and correspond to positions 22,061,474 to 22,061,658 and 22,130,089 to 22,130,330 on the chromosome 9 sequence. The expected size of the targeted genomic fragment is 68,856 bp. Hook2A is located 1,066 bp downstream of hook1A. Hook2B is located 776 bp upstream of hook1B. The targeting sequences (hooks) were cloned into vector pVC604 in orientations corresponding to their orientations in the human genome sequence (version hg19). The TAR vectors were linearized with ClaI (the site is located between the targeting sequences) before transformation to yield a molecule bounded by the desired region(s) sequences. Detailed physical maps of the TAR vectors are shown in Supplementary Figure 1.

### Yeast strain and transformation

A general scheme of TAR cloning is presented in Supplementary Figure 2A. For transformations, the highly transformable *Saccharomyces cerevisiae* strain VL6–48 (MATα, his3-Δ200, trp1-Δ1, ura3-52, lys2, ade2-101, met14) that has HIS3 deleted was used [43]. For TAR cloning, 2 to 3 μg of high molecular weight human genomic DNA were prepared, mixed with a ClaI-linearized TAR vector (1 μg), and presented to freshly prepared yeast spheroplasts. Yeast transformants were selected on synthetic complete medium plates lacking histidine. The yield of His^+^ transformants per 2–3 μg of genomic DNA using 1 μg of the TAR vector and 5 × 10^8^ spheroplasts varied between 10 and 60 colonies per plate. To identify desired CAD region-containing clones, the transformants were combined into pools and examined with the diagnostic primers (Supplementary Table 1) for the unique sequences not present in the TAR vectors but specific for the targeted genomic region. With DNA isolated from the C087 cells using the 9p21-1 and 9p21-2 TAR vectors the total number of transformants examined was 360. The transformants were organized into 12 pools, each containing 30 transformants. Eight pools were CAD-region-positive. In each pool, one individual transformant was CAD-region-positive. To identify which allele was cloned, we examined three TAR clones by PCR using pairs of primers specific for the mutated allele of the patient (three SNPs are characteristic for the mutant allele of the C087 patient, i.e. rs10757278, rs1333049, rs2383207) (Supplementary Table 1). Two transformants from the C087 patient were proven to be mutant after sequencing of PCR products. TAR cloning from normal genomic DNA (Promega) was carried out using the TAR vector 9p21-1. Three CAD-region-positive clones among 270 transformants analyzed were identified by a set of PCR reactions using pairs of diagnostic primers (Supplementary Table 1).

### Conversion of TAR/YAC isolated clones into a BAC form

A general scheme of retrofitting of a TAR/YAC molecule into a BAC molecule is presented in Supplementary Figure 2B. A retrofitting vector either BRV1 [52] or JH-BRV1 [53] contains the two short targeting hooks (300 bp each), separated by the unique BamHI site, that flank the ColE1 origin of replication in the pVC604-based TAR cloning vector. The hooks are homologous to the vector sequences of pVC604. Recombination of a BamHI-linearized retrofitting vector with a TAR/YAC vector part in yeast leads to replacement of the ColE1 origin of replication in the TAR cloning vector by a cassette containing the F’ factor origin replication, the chloramphenicol acetyltransferase (Cm^R^) gene, and the URA3 yeast selectable marker. A standard lithium acetate transformation procedure was used for retrofitting of YACs into BACs. YAC retrofitting was highly efficient: more than 95% of Ura^+^His^+^ transformants contained retrofitted YACs. The YAC/BACs were moved from yeast to *Escherichia coli* by electroporation. In brief, yeast chromosome-size DNAs were prepared in agarose plugs and, after melting and agarase treatment, the DNAs were electroporated into DH10B competent cells (Gibco/BRL) by using a Bio-Rad Gene Pulser as previously described [52].

### Physical characterization of YAC/TAR clones

Several approaches were taken to characterize the cloned material in a YAC form. To prove the presence of the predicted genomic sequences in YAC isolates, DNA from the yeast clones was examined by PCR with pairs of overlapping primers (Supplementary Table 1) covering the entire CAD region. To check the size of the YAC inserts, Southern blot hybridization was performed with a ^32^P-labeled probe. Specifically, genomic yeast DNA from CAD-positive clones was prepared in agarose plugs and exposed to a low dose of γ-rays (30 krad) to linearize circular YAC molecules [52]. The irradiated DNA was CHEF separated, and blot hybridized with a 125 bp yeast CEN6 probe. CEN6 sequence was amplified from the TAR vector using a unique pair of primers (Supplementary Table 1). Blots were incubated for 2 hrs at 65°C in prehybridization Church's buffer (0.5 M Na-phosphate buffer containing 7% SDS and 100 μg/ml of unlabeled salmon sperm carrier DNA). The labeled probe was heat denatured in boiling water for 5 min and snap cooled on ice. The probe was added to the hybridization buffer and allowed to hybridize overnight at 65 °C. Blots were washed twice in 2 × SSC (300 mM NaCl, 30 mM sodium citrate, pH 7.0), 0.1% SDS for 30 min at room temperature, then three times in 0.1× SSC, 0.1% SDS for 30 min at 65 °C. Blots were exposed to X-ray film for 24 hrs at −70 °C.

### Physical characterization of BAC clones

Several approaches were taken to characterize the TAR-cloned material in a BAC form. To check the size of the cloned inserts in the CAD-region-positive BAC clones, BAC DNA was digested with NotI that cuts only in the vector part, separated by clamped homogeneous electrical field electrophoresis (CHEF), and stained with EtBr. To prove the identity of TAR isolates, BAC DNA was digested by HindIII and run in 1% agarose gel. The ends of the BAC inserts were sequenced using specific primers (Supplementary Table 1). To demonstrate the presence of segmental duplications in the TAR isolates, the BAC DNA was digested either by AgeI or PmeI or FspI endonucleases that are present only once in the CAD interval.

### Analysis of junction between duplications

The junction sequence between duplications within the CAD interval was confirmed by PCR reaction using specific pairs of primers (Supplementary Table 1). The PCR products were blunt end cloned into pBluescipt II plasmid (Stratagene) and then sequenced using vector primers (Supplementary Table 1). All sequences were aligned and categorized.

### Construction of the vector V231

Construction of the V231 vector, which was used to re-clone the 50 kb repeats within the BAC A218, is illustrated in Supplementary Figure 5. Primers B095 and B102 (Supplementary Table 1) were used to amplify a subsection of the TAR-BRV-tTA^VP64^ plasmid [54], which included the BAC backbone, Chloramphenicol resistance gene (Cm^R^) and the PmeI-AscI-BamHI-AsiSI polylinker. The B095/B102 PCR product was then circularized to produce the plasmid A225. The plasmid A225 was then linearized with SacI and AscI. In parallel, the primers B635 and B629 (Supplementary Table 1) were used to PCR amplify the pBlueScript II KS plasmid (Stratagene) and add a polylinker. The resulting PCR fragment was digested with SacI and AscI. Then the linearized A225 plasmid and the SacI/AscI-digested PCR product were ligated to produce the vector V231.

### Sub-cloning of 50 kb repeats from the CAD-containing BAC

The general scheme of sub-cloning of the BAC regions is shown in Figures 4. First, DNA isolated from the CAD-containing BAC A218 (from a patient-derived cell line) (Figure 4A) was digested either by PmeI or AatII and then re-ligated. The ligation mixtures were electroporated into the ElectroMax DH10B bacterial cells (Life Technologies, Cat. No. 18290015). The Clm^R^ colonies were screened by three rounds of PCR reaction: i) by the primers specific for the yeast HIS3 gene (a positive control), ii) by the primers specific for the sequences around a PmeI site (a positive control) and iii) by the primers specific for the junction sequence, B578/B586, (a negative control) (Supplementary Table 1) to select the desirable clones for Next-Generation Sequencing (Figure 4B). Second, DNA from BAC A218 was digested by AgeI and run on CHEF. Then the fragments of ∼50 kb in size corresponding to duplicated units were gel-isolated and ligated into the vector V231. The ligation mixture was electroporated into the ElectroMax DH10B bacterial cells (Life Technologies, Cat. No.18290015). The Clm^R^ colonies were screened by PCR reaction using the primers specific for the junction between duplications (Supplementary Table 1). BACs negative for junction sequence were selected and used for Next-Generation Sequencing.

### Southern-blot hybridization analysis

Southern-blot hybridization was performed with a ^32^P-labelled probe as described previously [44, 52] with minor changes. Genomic DNA was prepared in agarose plugs and restriction-digested by NotI in the buffer recommended by the manufactory. The digested DNA was CHEF (CHEF Mapper, Bio-Rad) separated (autoprogram, 5-300 kb range, 16 hrs transfer), transferred to membrane (Amersham Hybond-N+) and blot-hybridized with a 862-bp probe specific for the CAD interval sequence in the 9p21.3 region. DNA sequence for the probe was amplified by PCR using the primers indicated in Supplementary Table 1. Blots were incubated for 2 hrs at 65°C in pre-hybridization Church's buffer (0.5 M Na-phosphate buffer containing 7% SDS and 100 μg/ml of unlabelled salmon sperm carrier DNA). The labeled probe was heat denatured in boiling water for 5 min and snap cooled on ice. The probe was added to the hybridization buffer and allowed to hybridize overnight at 65°C. Blot was washed twice in 2× SSC (300 mM NaCl, 30 mM sodium citrate, pH 7.0), 0.05% SDS for 10 min at room temperature, then twice in 2× SSC, 0.05% SDS for 5 min at 60°C, twice in 0.5× SSC, 0.05% SDS for 5 min at 60°C and twice in 0.25× SSC, 0.05% SDS for 5 min at 60°C. Blot was exposed to X-ray film for 24–72 hrs at −80°C.

### Determination of the copy number of SDs by quantitative real-time PCR

Three regions within the CAD interval were chosen for analysis. Two regions correspond to 5’ and 3’ ends of the 50 kb repeated unit. The third region is outside of the repeated unit and was used as the first control for copy number. The TaqMan probe and primers were designed using the PrimerQuest^®^ Design Tool (IDT Inc.), following the criteria indicated in the program. The specific TaqMan 9p21_5’ *27 bp* probe complementary to the analyzed region was designed (Supplementary Table 1). The 9p21_5’ probe contains a fluorophore FAM as a reporter. 9p21_5’ forward and reverse primers were created from the region on chromosome 9 (positions 22078776 to 22078919 in hg19). The size of the amplicon is 144 bp. The probe and primers were provided by IDT Inc. as a pre-mixed assay, which was diluted with sterile 1x TE buffer till 20x working concentration. RNAaseP kit was used as an internal reference (Applied Biosystems). The kit contains 20xRNAaseP mix with a VIC-labeled probe and specific primers for the RNAaseP gene used as the second control for copy number. As an additional control and double check, another set of probes was synthetized. The specific TaqMan 9p21_3’ probe 24 bp in size complementary to the analyzed region (chr9: positions 22117414 to 22117550 in hg19) and the specific TaqMan 9p21_control probe 23 bp in size complementary the unique DNA sequence present only in one copy per haploid genome (chr9: positions 22062099 to 22062185 in hg19) were designed. The 9p21_3’ probe contains a fluorophore 5′ FAM as a reporter. The size of the amplicon is 137 bp. The 9p21_control probe contains a fluorophore HEX/ZEN as a reporter. The size of the amplicon is 87 bp. Primer sequences and additional information are described in Supplementary Table 1. The samples were analyzed separately with set 1 (*9p21_5’* and RNAaseP) and set 2 (*9p21_3’* and *9p21_control*). In control experiments, amplification efficiency was close to 100% for all amplicons, signifying that both reactions proceeded with very high efficiencies. qPCR reactions were carried out using an CFX Connect (Bio-Rad) in a 96-well optical plate with a final reaction volume of 20 μl. All reactions in each plate were prepared from a single PCR Mastermix consisting of 2×iTaq Universal Probes Super Mix (Bio-Rad), 20×9p21_5’ Mix, 20×RNAaseP Mix, and HPLC pure water. A total of 10 ng of DNA template (5 μl) was dispensed into each of three sample wells for triplicate reactions. Each sample was run in triplicates to quantify the CAD analyzed region compared to the control probe sequences. Thermal cycling conditions included a pre-run of 3 min at 95°C. Cycle conditions were 40 cycles at 95°C for 10 sec and 60°C for 30 sec, according to the CFX 2 Step PCR Amplification Protocol (Bio-Rad). The relative copy number of *repeated units* was calculated for each sample using the comparative Pfaffl method.

### Determination of the copy number of SDs by droplet digital PCR

Droplet digital PCR was performed according to manufacturer's protocol (Bio-Rad). A specific FAM-labeled probe (dd9p21) against a region of interest was designed by Bio-Rad (Supplementary Table 1) and provider as ready-to-work 20x solution. As a control probe, we used Bio-Rad provided control against RPP30 which is present at one copy per cell. All reactions in each plate were prepared from a single PCR Mastermix consisting of ddPCR Supermix for Probes (Bio-Rad), dd9p21 Mix, RPP30 Mix, and HPLC pure water. A total of 10 ng of DNA template (5 μL) was dispensed into each of the three sample wells for triplicate reactions. Each sample was run in triplicates to quantify the 9p21.3 studied region compared with the internal RPP30 control gene. Bio-Rad provided “Quantasoft” software was used for data analysis. Reactions were performed with help of CCR Genomics Core (NIH/NCI).

### Illumina sequencing and library preparation

Sequencing libraries were prepared by tagmentation using the Nextera DNA Library Preparation Kit (Illumina Inc., San Diego, CA) according to the manufacturer's instructions. In short, 50 ng BAC DNA was tagged and fragmented using the Nextera transposome. The DNA was column purified, and amplified by suppression PCR introducing P5 and P7 ends with dual 8-nucleotide index sequences. Libraries were quantified by qPCR and sequenced on either MiSeq or NextSeq sequencers.

### Illumina alignment and variant calling

Alignments were performed with bwa version 0.7.12-r1039 [55] using the bwa mem algorithm against the hg19 reference genome. Duplicates were marked with picard version 1.92(1464) (https://github.com/broadinstitute/picard) using the MarkDuplicates tool. Variants from the reference genome were identified with freebayes version 0.9.21-7-g7dd41db [56] and non-SNVs were removed with vcftools version 0.1.14 [57].

## SUPPLEMENTARY MATERIALS FIGURES AND TABLES




