# Supplementary material for: Analysis of the 9p21.3 sequence associated with coronary artery disease reveals a tendency for duplication in a CAD patient
Source: Oncotarget. 2018 Feb 26;9(20):15275–91. doi: 10.18632/oncotarget.24567 (PMC5880603; doi:10.18632/oncotarget.24567)
Supplement: Supplementary file 1 [file oncotarget-09-15275-s001.pdf]

Analysis of the 9p21.3 sequence associated with coronary artery disease reveals a tendency for duplication in a CAD patient

SUPPLEMENTARY MATERIALS

A

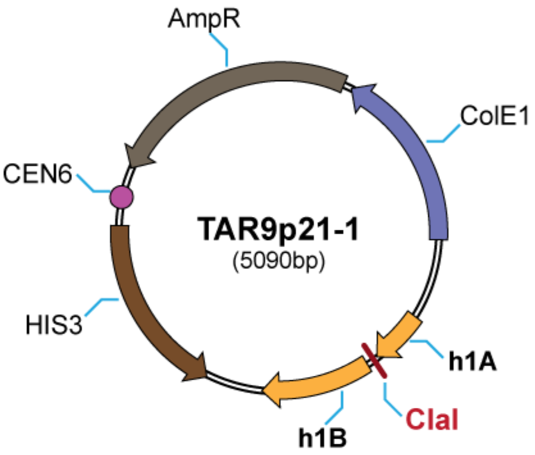

Hook	Size	Position in Genome Feb2009 (GRCh37/hg19)
h1A	171bp	Chr9: 22,062,540 - 22,062,710
h1B	300bp	Chr9: 22,129,014 - 22,129,313

B

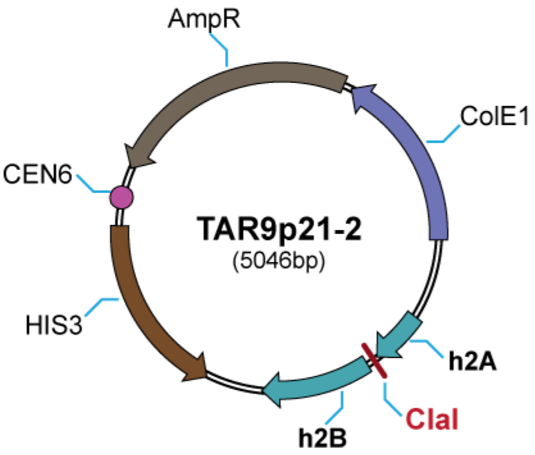

Hook	Size	Position in Genome Feb2009 (GRCh37/hg19)
h2A	185bp	Chr9: 22,061,474 - 22,061,658
h2B	242bp	Chr9: 22,130,089 - 22,130,330

Supplementary Figure 1: Map of the TAR vectors and the relative positions of hooks used to TAR clone the CAD interval. The hooks of TAR9p21-2 **(A)** are 776 bp upstream from the 5' end and 1066 bp downstream from the 3' end of the hooks in TAR9p21-1 **(B)**.

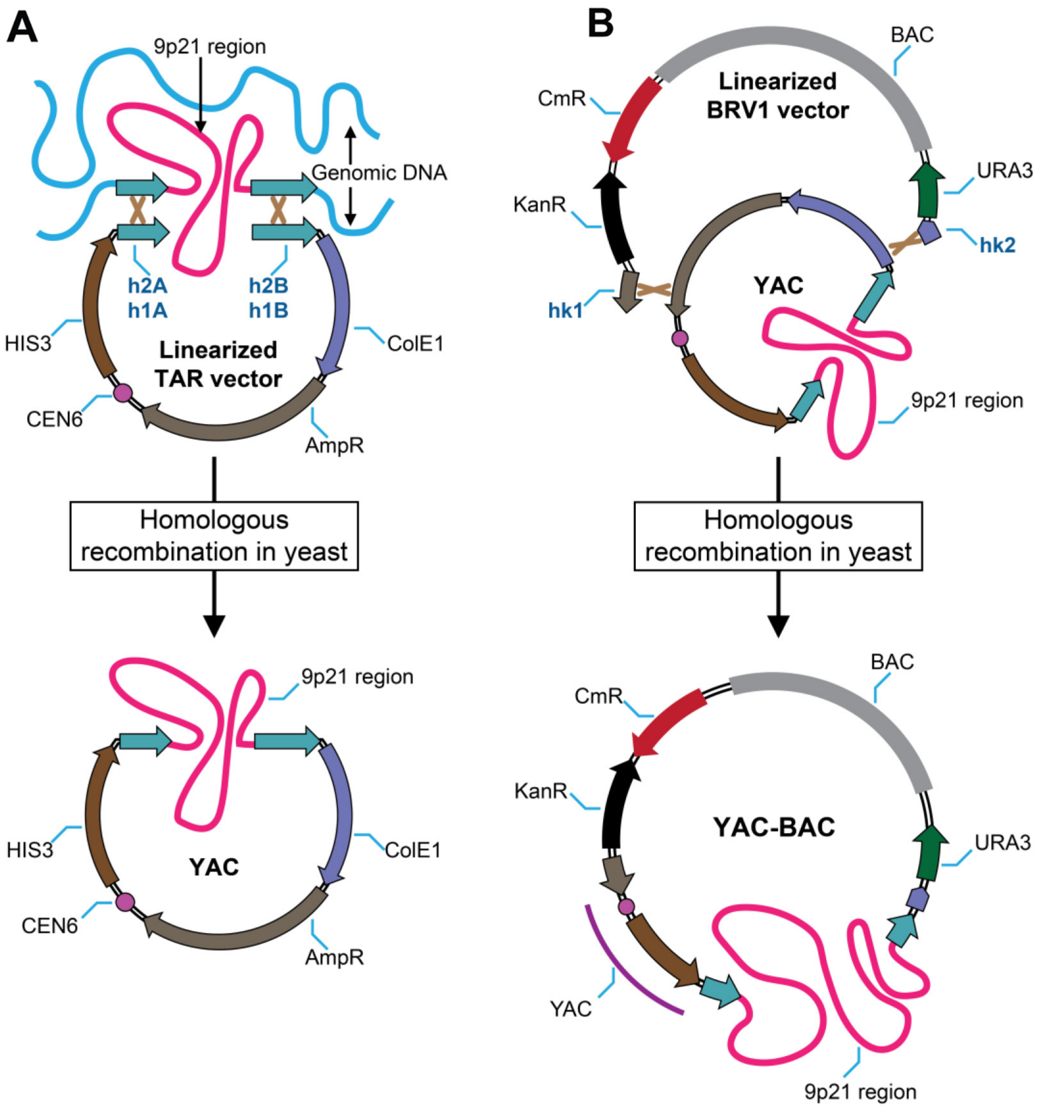

Supplementary Figure 2: Schematic diagram of **(A)** TAR cloning of the CAD interval via homologous recombination in yeast between the TAR vector and genomic DNA to form a YAC. **(B)** Retrofitting of a YAC with BRV1 plasmid to form a YAC-BAC molecule.

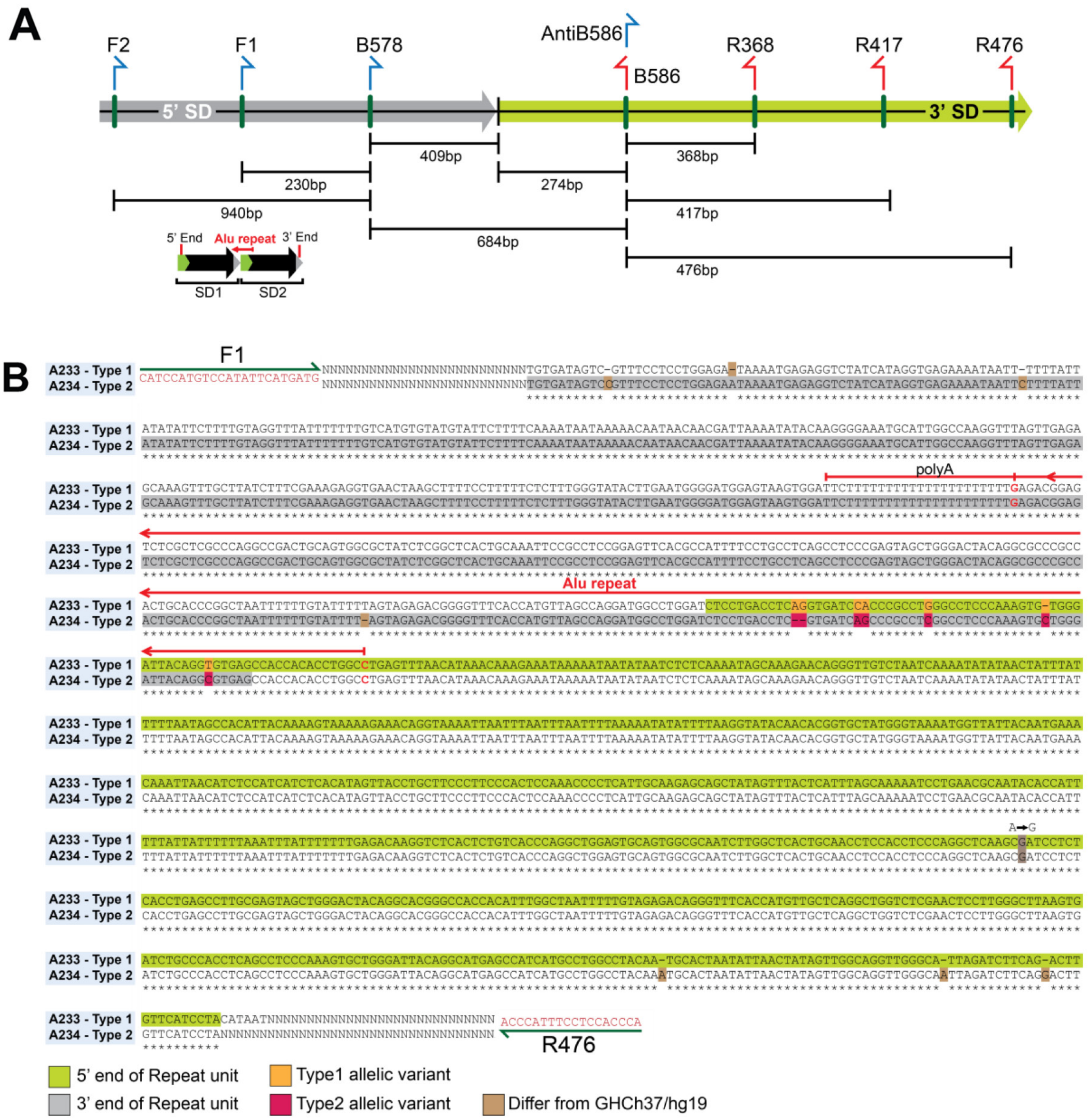

Supplementary Figure 3: (A) Relative position of primers used to PCR amplify longer junction products between CAD segmental duplication **(B)** The extended junction sequence reads between primer F1 and R476 (1.3kb) from BACs A233 and A234, which contain AgeI sections of the SD. BAC A233 has Type 1 junction while A234 has Type 2 junction.

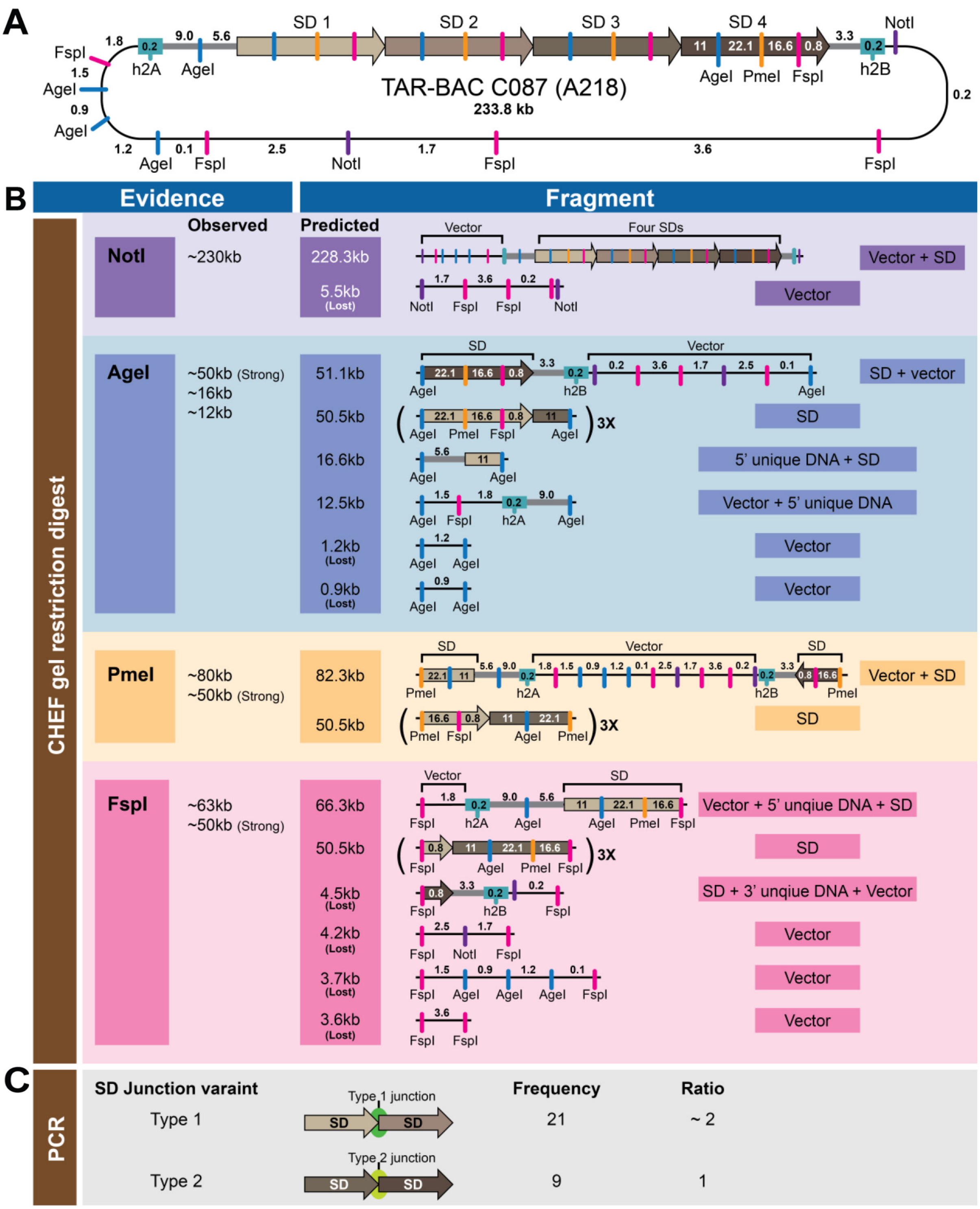

Supplementary Figure 4: **(A)** Proposed structure C087 TAR/BAC (A218) and evidence to support it. **(B)** Banding profile obtained restriction digest of A218. **(C)** The type and frequency of SD junctions amplified by primer B578/B586. "Lost" means not seen on the gel.

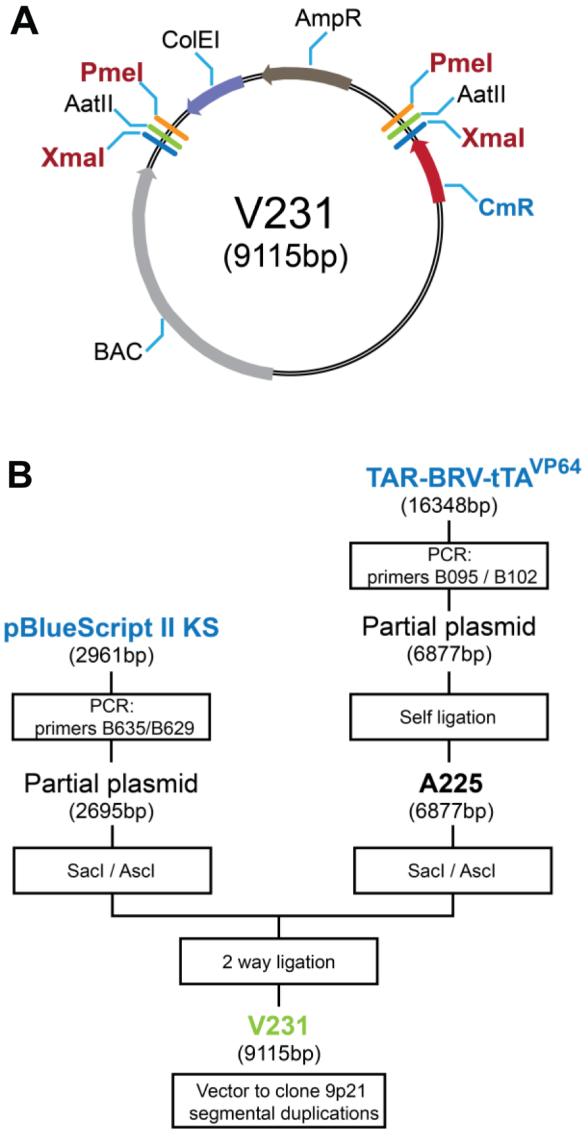

Supplementary Figure 5: **(A)** Map of the plasmid V231 and **(B)** flow diagram of V231 construction.

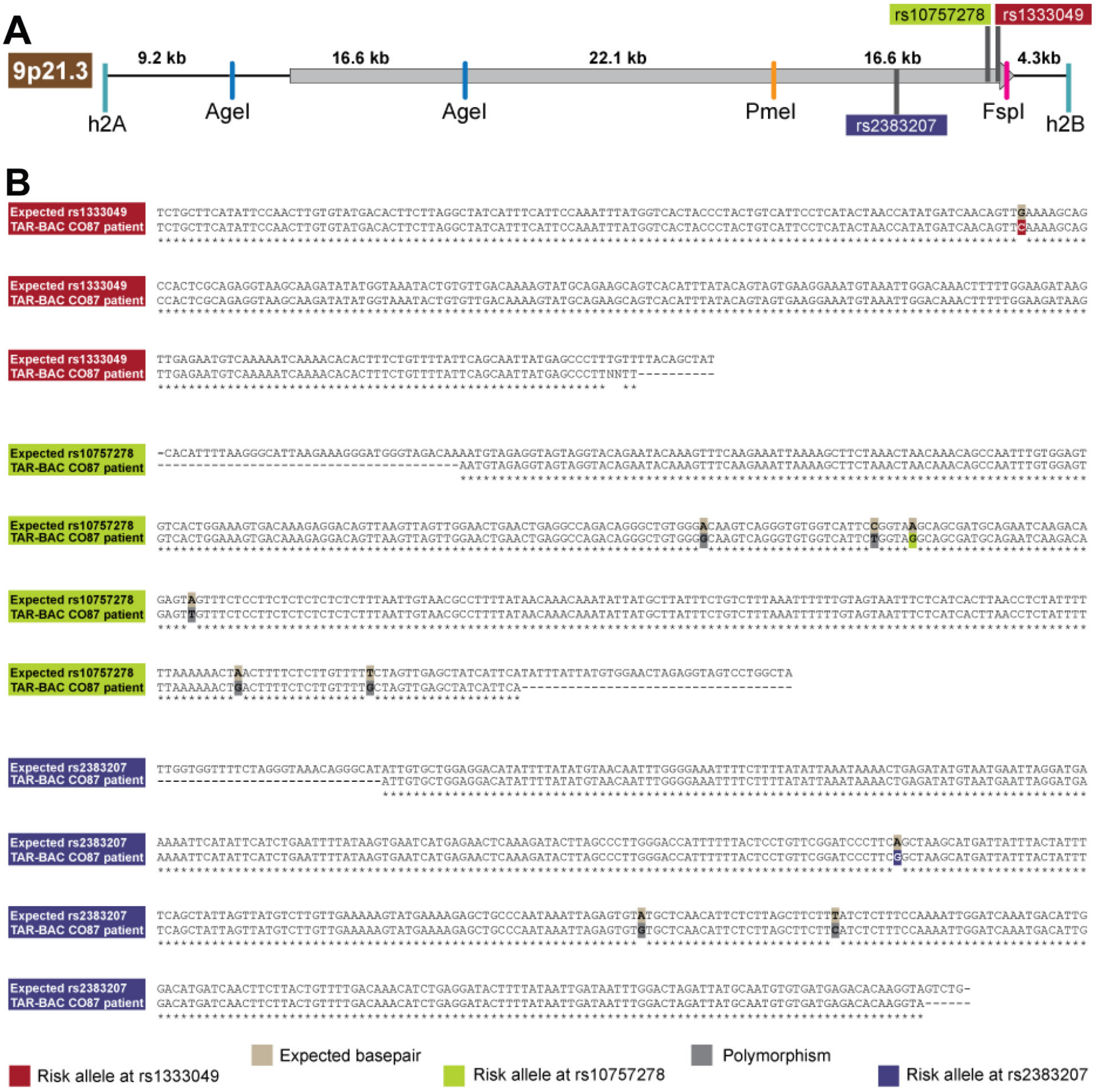

Supplementary Figure 6: Diagram **(A)** indicating the relative positions of the CAD disease associated alleles in the 9p21.3 locus. **(B)** Disease alleles (Red – position of rs1333049, Green – position of rs10757278, Blue – position of rs2383207) were found in the CO87 patient TAR/BAC. Disease alleles were not found in TAR/BAC derived from HeLa. In addition to the disease alleles, several other polymorphisms were found in the CO87 TAR/BAC.

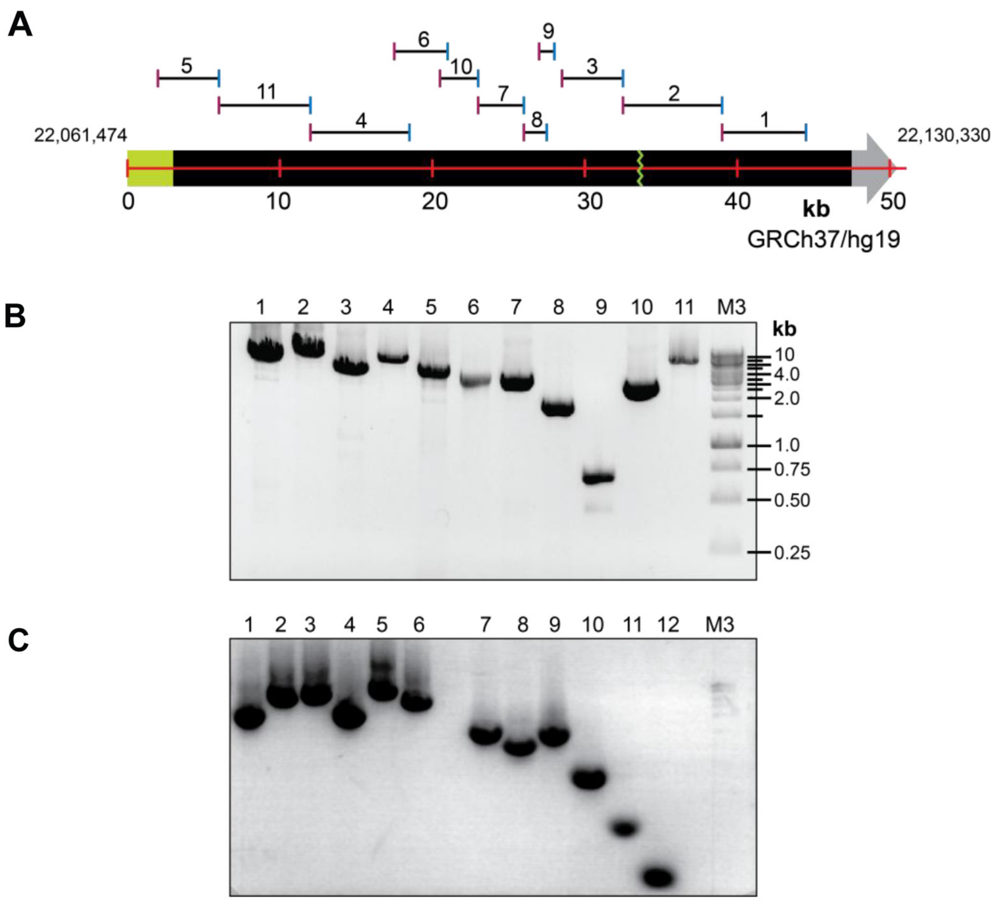

Supplementary Figure 7: (A) Relative positions of overlapping PCRs within the CAD region. **(B)** The overlapping PCRs covering the Promega derived TAR/BAC clone #1. Lane 1 corresponds to 51F/59R primers; lane 2 – 51R/46F; lane 3 – 46R/42F; lane 4 – 33R/27F; lane 5 – 20R/17F; lane 6 – 35R/33F; lane 7 – 40R/38F; lane 8 - 41R/40F; lane 9 – New41F/New41R; lane 10 – 38R/35F; lane 11 – 27R/20F. **(C)** The overlapping PCRs covering the Promega derived TAR/BAC clone #2. Lane 1 corresponds to 17F/20R primers; lane 2 - 20F/27R; lane 3 – 33R/27F; lane 4- 46R/42F; lane 5 - 51R/46F; lane 6 – 51F/59R; lane 7 – 33F/35R; lane 8 – 33F/38R; lane 9 – 38F/40R; lane 10 – 40F/41R; lane 11 – 42F/42R; lane 12 – New41F/New41R. All primers are in Supplementary Table 1.

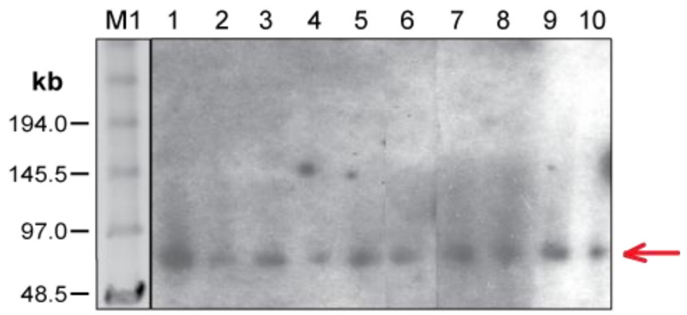

Supplementary Figure 8: Southern blot hybridization of YAC clones after transformation of A226 BAC into yeast cells. Genomic DNA from 10 His^{+} transformants were digested by NotI, separated by CHEF, and blot-hybridized with a 862bp specific probe (positions on chromosome 9: 22084459-22085320 in hg19). M1 - CHEF DNA Size Lambda Ladder (BIO-RAD).

Supplementary Table 1: Primers used in this study

See Supplementary File 1

Supplementary Table 2: SNPs detected from TAR/BAC A218 (C087 patient)

Chromosome	Position	Original	SNP	Chromosome	Position	Original	SNP
chr9	22064391	G	A	chr9	22098574	A	G
chr9	22064465	A	G	chr9	22098619	A	G
chr9	22065002	C	G	chr9	22099247	A	C
chr9	22065620	A	G	chr9	22099568	C	A
chr9	22065657	G	C	chr9	22100176	G	C
chr9	22066363	T	A	chr9	22102165	C	T
chr9	22067004	G	A	chr9	22103183	G	T
chr9	22067276	T	A	chr9	22103341	T	G
chr9	22067542	C	T	chr9	22103813	A	G
chr9	22067554	C	T	chr9	22105927	T	C
chr9	22067593	A	G	chr9	22106225	G	A
chr9	22067830	G	A	chr9	22106271	A	G
chr9	22068652	G	A	chr9	22106731	T	A
chr9	22072264	A	G	chr9	22110131	T	C
chr9	22072301	G	C	chr9	22112241	A	C
chr9	22072638	G	A	chr9	22112427	A	G
chr9	22072719	A	G	chr9	22112599	T	C
chr9	22073996	T	G	chr9	22114469	G	C
chr9	22076071	T	C	chr9	22114495	A	G
chr9	22076795	A	G	chr9	22115026	A	G
chr9	22077085	A	C	chr9	22115286	T	C
chr9	22077543	C	T	chr9	22115589	A	T
chr9	22081397	G	T	chr9	22115959	A	G
chr9	22081850	C	T	chr9	22116046	A	G
chr9	22082375	A	C	chr9	22116071	T	C
chr9	22083404	C	T	chr9	22116220	T	C
chr9	22084310	C	T	chr9	22119195	T	C
chr9	22085598	T	C	chr9	22121349	A	T
chr9	22085876	T	C	chr9	22123766	A	C
chr9	22087473	T	C	chr9	22124123	T	A
chr9	22088260	C	T	chr9	22124140	A	T

(Continued)

Chromosome	Position	Original	SNP	Chromosome	Position	Original	SNP
chr9	22090521	G	A	chr9	22124450	A	G
chr9	22090603	C	T	chr9	22124472	C	T
chr9	22091069	C	T	chr9	22124477	A	G
chr9	22091924	G	T	chr9	22124504	A	T
chr9	22092257	G	A	chr9	22124630	A	G
chr9	22092924	A	G	chr9	22124744	C	G
chr9	22093299	A	T	chr9	22125347	A	C
chr9	22094330	A	G	chr9	22125503	G	C
chr9	22094796	A	C	chr9	22125913	C	T
chr9	22096055	A	G	chr9	22128709	C	G

Supplementary Table 3: Types and junctions found in various TAR/BACs and genomic C087 DNA

C087 TAR/BAC – PCR B586/B578		
**Type**	**Variant**	**Number***
Type 1	Same as expected sequence	21
Type 2	Same as expected sequence	9
	**Total**	30

C087 genomic DNA – PCR B586/B578		
**Type**	**Variant**	**Number**
Type 1	Same as expected sequence	4
Type 1	482C>T,	1
Type 1	397C>T, 419G>C	1
Type 1	518G>A	1
Type 2	Same as expected sequence	7
Type 2	252T>C; 397C>A	1
	**Total**	15

Supplementary Table 4: Analysis of the number of SD at the CAD interval in normal individuals by ddPCR*

Population	Total analyzed number	Samples with 1 Copy	Samples with 2 Copy	Samples with 3 Copy
Caucasian	83	83	0	0

*The number is given per haploid genome.
