# Supplementary material for: Analysis of the 9p21.3 sequence associated with coronary artery disease reveals a tendency for duplication in a CAD patient
Source: Oncotarget. 2018 Feb 26;9(20):15275–91. doi: 10.18632/oncotarget.24567 (PMC5880603; doi:10.18632/oncotarget.24567)
Supplement: Supplementary file 2 [file oncotarget-09-15275-s002.doc]

**Supplementary Table S1** Primers used in this study

| **TAR-9p21-1** |  | **Size** |
| --- | --- | --- |
|  |  |  |
| **5’ hook h1A** |  |  |
| TAR 9XhoF | 5’ TTAATGGGGCTACATGAGGA 3’ | 171bp |
| TAR 9XhoR | 5’ CAGACACCCCTCAGCCTCC 3’ |  |
|  |  |  |
| **3’ hook h1B** |  |  |
| TAR 9SpeF | 5’ GTCTGGAATTACTCCAGTTAATCTTGG 3’ | 299bp |
| TAR 9SpeR | 5’ ATAGCAGAAGCTGCAAAATCCAC 3’ |  |
|  |  |  |

| **TAR-9p21-2** |  | **Size** |
| --- | --- | --- |
|  |  |  |
| **5’ hook h2A** |  |  |
| TAR 9pXhoF | 5’TGGGCCTATTAGCTACAAACATG 3’ | 185bp |
| TAR 9pXhoR | 5’CTGAGTATTAATTCCTGTTTCCAAATAG 3’ |  |
|  |  |  |
| **3’ hook H2B** |  |  |
| TAR 9pSpeF | 5’CAGCAGCCTGTACTTTACTCCAAC 3’ | 242bp |
| TAR 9pSpeR | 5’TTGCAGAAGGAGACAGAATTCTC 3’ |  |
|  |  |  |

| **Diagnostic primers** |  | **Size** |
| --- | --- | --- |
|  |  |  |
| **For 5’ end** |  |  |
| Diag F1 | 5’ CCAAAGGGAGGAAGGTCAAT 3’ | 187bp |
| Diag R1 | 5’ GACAAATCCTACTTCAGACCCTCA 3’ |  |
|  |  |  |
| **For 3’ end** |  |  |
| Diag F2 | 5’ CCTTTCTCACAGCTGGTGAA 3’ | 252bp |
| Diag R2 | 5’ATCCTCGTTAAGTCAAAGTTCGTA 3’ |  |
|  |  |  |

| **Internal part of TAR clones** | | **Size** |
| --- | --- | --- |
|  |  |  |
| 13 F | 5’ AAGCGCCAATCTATCTGTGACT 3’ | 344bp |
| 13 R | 5’ TCCTGTCCTCACCCTTCTTTGT 3’ |  |
|  |  |  |
| 20 F | 5’ ATCAGGTGTAAGCCACGGAA 3’ | 171bp |
| 20 R | 5’ ACATGTCCTCTTCCTGCTACCA 3’ |  |
|  |  |  |
| 35 F | 5’ GGTGGAGAACTTCAGTAGAGGAAGT 3’ | 314bp |
| 35 R | 5’ AGCTAGTGGTATAAGGAGGTGATGTG 3’ |  |
|  |  |  |
| 41 F | 5’ CATATGGCATTGCCATATCGT 3’ | 344bp |
| 41 R | 5’ CATGTAGAGAGCATCCTCACTTG 3’ |  |
|  |  |  |
| 51 F | 5’ GACCTGTGCTCCCATTAAGGT 3’ | 293bp |
| 51 R | 5’ GAAGGGATCCGAACAGGAGT 3’ |  |
|  |  |  |

| **Overlapping primers** | | **Size** |
| --- | --- | --- |
|  |  |  |
| New41 F | 5’ TCAGCCAGCATATCTGTAGCTT 3’ | 678bp |
| New41 R | 5’ AGGAGAGGTACGGTGGTAGAGA 3’ |  |
|  |  |  |
| New41 F | 5’ TCAGCCAGCATATCTGTAGCTT 3’ | 523bp |
| 41 R | 5’ CATGTAGAGAGCATCCTCACTTG 3’ |  |
|  |  |  |
| 41 F | 5’ CATATGGCATTGCCATATCGT 3’ | 399bp |
| New41 R | 5’ AGGAGAGGTACGGTGGTAGAGA 3’ |  |
|  |  |  |
| 17 F | 5’ AGCTGAGGTTTGGGTATGCAG 3’ | 4,204bp |
| 20 R | 5’ ACATGTCCTCTTCCTGCTACCA 3’ |  |
|  |  |  |
| 20 F | 5’ ATCAGGTGTAAGCCACGGAA 3’ | 6,137bp |
| 27 R | 5’ GCTGGACAAGCAGGTGTGT 3’ |  |
|  |  |  |
| 27 F | 5’ GTGGAGTTCACATGAGTCCCTT 3’ | 6,332bp |
| 33 R | 5’ TACCCCTCTGAGACGAAGCTT 3’ |  |
|  |  |  |
| 42 F | 5’ ACTGCAACAGCTACACTGACTCTT 3’ | 4,049bp |
| 46 R | 5’ TCAGGTGACATGACCTGCA 3’ |  |
|  |  |  |
| 46 F | 5’ CAGTGAGTGAATAACTGATGAGGTC 3’ | 7,203bp |
| 51 R | 5’ GAAGGGATCCGAACAGGAGT 3’ |  |
|  |  |  |

| **Overlapping primers** | | **Size** |
| --- | --- | --- |
|  |  |  |
| 51 F | 5’ GACCTGTGCTCCCATTAAGGT 3’ | 5,623bp |
| 59 R | 5’ ACCTCTACCCATCTACCCATCAC 3’ |  |
|  |  |  |
| 33 F | 5’ TTGCTAGATTGGGGAAGTTCTC 3’ | 3,203bp |
| 35 R | 5’ AGCTAGTGGTATAAGGAGGTGATGTG 3’ |  |
|  |  |  |
| 35 F | 5’ GGTGGAGAACTTCAGTAGAGGAAGT 3’ | 2,687bp |
| 38 R | 5’ CTACCGCTGTTCTCCCTCAG 3’ |  |
|  |  |  |
| 38 F | 5’ TCCAGGCAAGAAATACTGAGATG 3’ | 3,129bp |
| 40 R | 5’ ACATCTTTCCTGCTCTCTGCAC 3’ |  |
|  |  |  |
| 40 F | 5’ TCCTTTAGTCTCTCCTTTAGGCTGT 3’ | 1,805bp |
| 41 R | 5’ CATGTAGAGAGCATCCTCACTTG 3’ |  |
|  |  |  |
| 41 F | 5’ CATATGGCATTGCCATATCGT 3’ | 1,094bp |
| 42 R | 5’ TGCACTGAAACCTCCAACACTA 3’ |  |
|  |  |  |
| 6500 F | 5’ TGGGTAGGTCATACAGAGCGATA 3’ | 6,804bp |
| 13 R | 5’ TCCTGTCCTCACCCTTCTTTGT 3’ |  |
|  |  |  |
| 13 F | 5’ AAGCGCCAATCTATCTGTGACT 3’ | 3,422bp |
| 17 R | 5’ GACTTGTCCCCATCTCTCTGA 3’ |  |
|  |  |  |
| 6500 F | 5’ TGGGTAGGTCATACAGAGCGATA 3’ | 9,882bp |
| 17 R | 5’ GACTTGTCCCCATCTCTCTGA 3’ |  |
|  |  |  |
| 38 F | 5’ TCCAGGCAAGAAATACTGAGATG 3’ | 4,776bp |
| 41 R | 5’ CATGTAGAGAGCATCCTCACTTG 3’ |  |
|  |  |  |
| New41 F | 5’ TCAGCCAGCATATCTGTAGCTT 3’ | 678bp |
| New41 R | 5’ AGGAGAGGTACGGTGGTAGAGA 3’ |  |
|  |  |  |

| **Predicted junction between SDs** | | | | | **Size** |
| --- | --- | --- | --- | --- | --- |
|  |  | | |  |  |
| **Primer pairs that worked** | | | |  |  |
| B578 | | | Forward | 5’ TACAAGGGGAAATGCATTGG 3’ | 684bp |
| B577 | | | Forward | 5’ TTGAATGGGGATGGAGTAAGTG 3’ | 584bp |
| B586 | | | Reverse | 5’ TGGGAAGGGAAGCAGGTAAC 3’ |  |
|  |  | | |  |  |
| **Extended PCR junction** | | | | |  |
| F1 | | | Forward | 5’ CATCCATGTCCATATTCATGATG 3’ | 861bp |
| F2 | | | Forward | 5’ CACTCGCAGAGGTAAGCAAGAT 3’ | 1596bp |
| B586 | | | Reverse | 5’ TGGGAAGGGAAGCAGGTAAC 3’ |  |
|  | |  | |  |  |
| Anti-B586 | | | Forward | 5’GTTACCTGCTTCCCTTCCCA 3’ |  |
| R368 | | | Reverse | 5’ AGTGCATTGTAGGCCAGGC 3’ | 368bp |
| R417 | | | Reverse | 5’ ATCTAATGCCCAACCTGCC 3’ | 417bp |
| R476 | | | Reverse | 5’ TGGGTGGAGGAAATGGGT 3’ | 476bp |
|  | | |  |  |  |
| F1 | | | Forward | 5’ CATCCATGTCCATATTCATGATG 3’ |  |
| R368 | | | Reverse | 5’ AGTGCATTGTAGGCCAGGC 3’ | 1.2kb |
| R417 | | | Reverse | 5’ ATCTAATGCCCAACCTGCC 3’ | 1.3kb |
| R476 | | | Reverse | 5’ TGGGTGGAGGAAATGGGT 3’ | 1.3kb |
|  | | |  |  |  |
| **Primer pairs that did not work** | | | | |  |
| B578 | | | Forward | 5’ TACAAGGGGAAATGCATTGGC 3’ |  |
| B577 | | | Forward | 5’ TTGAATGGGGATGGAGTAAGTG 3’ |  |
| B581 | | | Reverse | 5’ TGCTTTCTAACCATTGTGAGGA 3’ |  |
| B580 | | | Reverse | 5’ TCTGTCAACTCCACTCCAACC 3’ |  |
| B579 | | | Reverse | 5’ TAGCACTTCACCATGTGCCA 3’ |  |
|  | | |  |  |  |

| **Primers for pathogenic SNPs** | | **Size** |
| --- | --- | --- |
|  |  |  |
| **rs1333049** |  |  |
| Rs049-F | 5’ TCTGCTTCATATTCCAACTTGTGT 3’ | 312bp |
| Rs049-R | 5’ ATAGCTGTAAAACAAAGGGCTCA 3’ |  |
|  |  |  |
| **rs10757278** |  |  |
| rs278-F | 5’ CACATTTTAAGGGCATTAAGAAAGG 3’ | 441bp |
| rs278-R | 5’ TAGCCAGGACTACCTCTAGTTCCA 3’ |  |
|  |  |  |
| **rs2383207** |  |  |
| rs3207-F | 5’ TTGGTGGTTTTCTAGGGTAAACAG 3’ | 464bp |
| rs3207-R | 5’ CAGACTACCTTGTGTCTCATCACAC 3’ |  |
|  |  |  |

| **Primers for sequences flanking SDs** | | **Size** |
| --- | --- | --- |
|  |  |  |
| **Upstream SDs** |  |  |
| ReverseSD +292 | 5’ TGGGAAGGGAAGCAGGTAAC 3’ |  |
| Up588F | 5’ GTACTGGCATCCTCAGATTGGA 3’ | 588bp |
| Up1284F | 5’ CCTGAGGAGATTGTAGGAGACCT 3’ | 1,285bp |
| Up1288F | 5’ CACACCTGAGGAGATTGTAGGA 3’ | 1,289bp |
| Up1302F’ | 5’ AATGATGAGGAATCACACCTGAG 3’ | 1,302bp |
|  |  |  |
| **Downstream SDs** |  |  |
| Forward -354 | 5’ TACAAGGGGAAATGCATTGG 3’ |  |
| Dn473R | 5’ TATGACACCTCCCCACCTTCT 3’ | 473bp |
| Dn1191R | 5’ CAGAGATCCTCAGTGGACACAG 3’ | 1,191bp |
| Dn1225R | 5’ CTCACTCTCATCCCTGCCTTC 3’ | 1,225bp |
|  |  |  |

| **Primers for construction of V231 plasmid** | | **Size** | |
| --- | --- | --- | --- |
|  |  | |  |
| B635 | 5’*GGATGAATGGCGCGCCCGGGACGTCAGTTTAAACATACGAGCCGGAAGCATAA 3’ | | 2961bp |
| B629 | 5’*GGATCAGAGCTCACCCGGGACGTCGTTTAAACAGCTGGCGTAATAGCGAAG 3’ | |  |
|  |  | |  |
| B095 | 5’ GGTGGTCTAGTGGTTAGGAT 3’ | | 6877bp |
| B102 | 5’ GGAAGGGAAGAAAGCGAAAG 3’ | |  |
|  |  | |  |

| **Primers for qPCR (9p21 assay)** | | **Size** |
| --- | --- | --- |
|  |  |  |
| **9p21_5’_Forvard** | 5’ TTCTCACTCTGTAAATCAGCTCTG 3’ | 144bp |
| **9p21_5’_Reverse** | 5’ TCCTCATTTTACCAGACAATGGG 3’ |  |
| **9p21_5’_Probe** | 5’ 56-FAM/CAAAACTCTGCATACCCAAACCTCAGC/ 36-TAMSp 3’ |  |
| **9p21_3’_Forvard** | 5’ TCTTAACCTGAAATTGAAAACCCTG 3’ | 137bp |
| **9p21_3’_Reverse** | 5’ CCAGTCCGAGATGTATGAATCC 3’ |  |
| **9p21_3’_Probe** | 5’ 56-FAM/TCCTCTCCCACGAGCTTCCAAATG/36-TAMSp 3’ |  |
|  |  |  |
| **9p21_control_Forvard** | 5’ GAAGGGAAGATACAGGTGGAAC 3’ | 87bp |
| **9p21_ control _Reverse** | 5’ TCTAGAACTGATAGGGAGCCAG 3’ |  |
| **9p21_ control _Probe** | 5’ /5HEX/TGGGCCAGT/ZEN/GTTTGCAGAGGA/3IABkFQ/ 3’ |  |
|  |  |  |

| **Primers for ddPCR (dd9p21 assay)** | | **Size** |
| --- | --- | --- |
|  |  |  |
| **dd9p21_Forvard** | 5’ AGGCCATCAGGGAGTTTCTT 3’ | 136bp |
| **dd9p21_Reverse** | 5’ GCAGCAAGTTCACCATCTCA 3’ |  |
| **dd9p21_Probe** | 5’ FAM/TGTCCTCTTCACCAGCCCAT 3’ |  |

| **Primers for Southern blot** | | **Size** |
| --- | --- | --- |
|  |  |  |
| CEN6-F | 5’ TTTTCATCACGTGCTATAAAAATA 3’ | 125bp |
| CEN6-R | 5’ CTTTTACATCTTCGGAAAACA 3’ |  |
|  |  |  |

| **Primers for Southern blot** | | **Size** |
| --- | --- | --- |
|  |  |  |
| **Sout3-F** | 5’ GTACTTCCCAGGGAGCAGAGTAT 3’ | 862bp |
| **Sout3-R** | 5’ GGTTGGGATAAGCATACCTTGAG 3’ |  |

| **Diagnostic primers for the HIS3 gene and PmeI site** | | **Size** |
| --- | --- | --- |
|  |  |  |
| His3 F2 | 5’ CATAGACGACCATCACACCA 3’ | 378bp |
| His3 R2 | 5’ ACATCGTTGGTACCATTGGG 3’ |  |
|  |  |  |
| Pm-F | 5’ GTCACCTGACAGAAACTTTGGAC 3’ | 710bp |
| DiagPme1R: | 5’ GAGAGCAAGGGAAAATGAGTG 3’ |  |
|  |  |  |
| Pm-F | 5’ GTCACCTGACAGAAACTTTGGAC 3’ | 1263bp |
| DiagPme2R | 5’ TGTATTCCAAGACTTCTGGGAATC 3’ |  |
|  |  |  |
